# A genome-wide association study for natural antibodies measured in blood of Canadian Holstein cows

**DOI:** 10.1186/s12864-018-5062-6

**Published:** 2018-09-21

**Authors:** Britt de Klerk, Mehdi Emam, Kathleen A. Thompson-Crispi, Mehdi Sargolzaei, Johan J. van der Poel, Bonnie A. Mallard

**Affiliations:** 1 Animal Breeding and Genomics Centre, Wageningen University, P.O. Box 338, Wageningen, The Netherlands; 2 Trouw Nutrition Canada Inc., 150 Research Lane, Suite 200, Guelph, ON N1G 4T2 Canada; 3 Centre for Genetic Improvement of Livestock, University of Guelph, Guelph, ON Canada; 4 Semex Alliance, Guelph, ON Canada; 5 Department of Pathobiology, Ontario Veterinary College, Genetic improvement of livestock, University of Guelph, Guelph, ON N1G 2W1 Canada

**Keywords:** Genome-wide-association study, Natural antibody, Dairy cattle, Immune system, Vesicle trafficking, Immunoglobulin class switching

## Abstract

**Background:**

Natural antibodies (NAb) are an important component of the innate immune system, and fight infections as a part of the first line defence. NAb are poly-reactive and can respond non-specifically to antigens. Therefore, NAb may be a key trait when evaluating an animal’s potential natural disease resistance. Variation in NAb is caused by both genetic and environmental factors. In this study genetic parameters of NAb were estimated and a genome-wide association study (GWAS) was performed to gain further understanding on the genes that are responsible for the observed genetic variation of NAb in Canadian Holsteins.

**Results:**

In total, blood samples of 1327 cows from 64 farms were studied. NAb binding to keyhole limpet hemocyanin (KLH) were determined via indirect ELISA. Immunoglobulin (Ig) isotypes, IgG and IgM, were evaluated. From the sample population, 925 cows were genotyped for 45,187 markers and each individual marker was tested to detect genetic variation in NAb levels. The relationships among animals was accounted for with genomic relationship. Results show heritabilities of 0.27 ± 0.064 (IgG) and 0.31 ± 0.065 (IgM). In total, 23 SNPs were found to be associated with IgG, but no SNPs were associated with IgM (FDR *p*-value < 0.05). The significant SNPs were located on autosomal chromosomes 1, 20 and 21 of the cow genome. Functional annotation analysis of the positional candidate genes revealed two sets of genes with biologically relevant functions related to NAb. In one set, seven genes with crucial roles in the production of antibody in B cells were associated with the trafficking of vesicles inside the cells between organelles. In the second set, two genes among positional candidate genes were associated with isotype class-switching and somatic hypermutation of B cells.

**Conclusions:**

This study demonstrated the possibility of increasing NAb through selective breeding. In addition, the effects of two candidate pathways are proposed for further investigation of NAb production in Holsteins.

**Electronic supplementary material:**

The online version of this article (10.1186/s12864-018-5062-6) contains supplementary material, which is available to authorized users.

## Background

Over the last several decades, breeding in dairy cattle mainly focused on production and fertility traits, with less emphasis on health traits. Health problems, however, can cause substantial economic losses to the dairy industry. The economic losses, together with the rising awareness of animal welfare, increased herd size, and less attention for individual animals, have led to a growing focus on health traits. Health parameters currently used in dairy cattle breeding programs consider milk quality related traits, including somatic cell count or score (SCC or SCS) and clinical mastitis resistance, as well as claw related health parameters, and metabolic disorders, such as ketosis. Somatic cell score is often used as a health indicator in breeding indices and has a heritability between 0.10 and 0.17, when based on lactation means [1–4]. Somatic cell score was associated with mastitis [5], and is therefore, related to udder-quality rather than the total health status of a cow.

Currently, national animal breeding indices do not select for overall health status based on immune capacity. Therefore, an opportunity exists to find immune response parameters with potential association with overall disease resistance. Natural antibodies (NAb) are a candidate for improving innate host defence in dairy cows. NAb is produced by B1-cells and this type of antibody (Ab) with broad specificity is a component of the innate immune system that can be found in animals without prior exposure to antigens [6]. In this way, NAb can play an important role in the first line of defence against different kinds of pathogens since they are polyreactive Ab that bind different conserved structures including carbohydrate, nucleic acid and phospholipids [7, 8]. Different NAb isotypes exist, with isotype IgM being most common, and isotypes IgG and IgA found to a lesser extent [9]. Additionally, NAb provide a link between the innate and adaptive immune system [10]. In dairy cows, NAb are measurable in both blood- and milk samples [11, 12].

Many studies have shown that it is possible to select for healthier cows based on adaptive immune response traits [13–17]. In these studies, the relationships between economically important diseases, including mastitis, metritis, pneumonia, displaced abomasum and retained placenta of dairy cows and their immune responses were examined and it was demonstrated that specific antibodies (SpAb), which are part of the adaptive immune system, are useful biomarkers for disease resistance. Cows were tested for two different adaptive immune responses, antibody-mediated (AMIR) and cell-mediated immune response (CMIR) [17]. A decreased incidence of mastitis was found for cows with a high AMIR, CMIR and overall immune response. Moreover, a higher incidence of metritis was found for cows with a low CMIR and cows with low overall immune response had an increased incidence of retained foetal membrane and displaced abomasum [17]. However, SpAb are only measured after immunization of the cows, whereas NAb can be measured prior to immunization, and therefore may have practical advantages in a breeding program.

Recently, NAb have been studied as a predictor for different diseases in dairy cows. NAb binding KLH and Lipopolysaccharides (LPS) in plasma were found to be negatively associated with metabolic health, but not statistically significant. In contrast, the level of these NAb in milk were positively associated with metabolic health, significantly [18, 19]. Associations between immune response and metabolism are not uncommon [20–23]. Van Knegsel et al. found a positive trend between clinical mastitis incidence and NAb binding to the self-antigen, myosin, and a negative trend between mastitis incidence and NAb binding to the self-antigen, transferrin [19]. Banos et al. hypothesized that higher NAb levels (binding to KLH) can be associated with improved capacity of the innate immune system to respond to pathogen challenges. However, they also found that a poorer nutritional status was related with higher NAb levels [24]. Together these studies suggested a potential association between NAb and incidence of infectious and metabolic diseases, but caution must be exercised since some NAb can be cross reactive binding to self antigens.

Variation in NAb stems from both genetic and environmental factors. Heritability estimates of NAb measured in blood or milk of dairy cows, ranges from 0.08–0.45 [11, 25–27], where IgM generally has the highest heritability estimates (0.18–0.45) and IgG has lower heritability estimates (0.08–0.31). Additionally, NAb measured in blood serum had higher heritability (0.15–0.25) compared to NAb measured in milk (0.08–0.23) [11]. The relatively high heritability (especially for the IgM isotype) shows potential for genetic selection.

The objective of this study was to identify regions of the bovine genome that are associated with the level of NAb of the IgM and IgG isotypes. Results are expected to provide a further step in unravelling the genetic control of NAb, which is, given the resemblances in NAb between species, not only relevant for dairy cattle but also for many other species.

## Results

In total, 925 genotyped animals were included in this analysis. The dataset contained 45,187 SNPs and after removing SNPs on X chromosome and with MAF less than 1%, the number of SNPs used for the final analyses was 43,471. Animals with deviating days in milk (> 500 days in milking) were removed from the total dataset (*n* = 6). Mean and the corresponding standard deviations for both IgG and IgM isotypes are shown in Table 1. Heritability based on genomic information for isotype IgG was estimated at 0.27 ± 0.064 and for isotype IgM heritability was estimated at 0.31 ± 0.065.

**Table 1:** Means and standard deviations for the corrected optical destiny of natural antibodies with immunoglobulin isotypes IgG and IgM with and without log-transformation; *n* =1327

Variable	Mean	Std dev.	Min	Max
IgG	0.07	7.58	0.00	18.45
IgM	1.15	2.01	0.11	9.79
IgG_log10	−1.17	0.88	−4.19	1.27
IgM_log10	0.06	0.30	−0.96	0.99

**Fig. 1:**
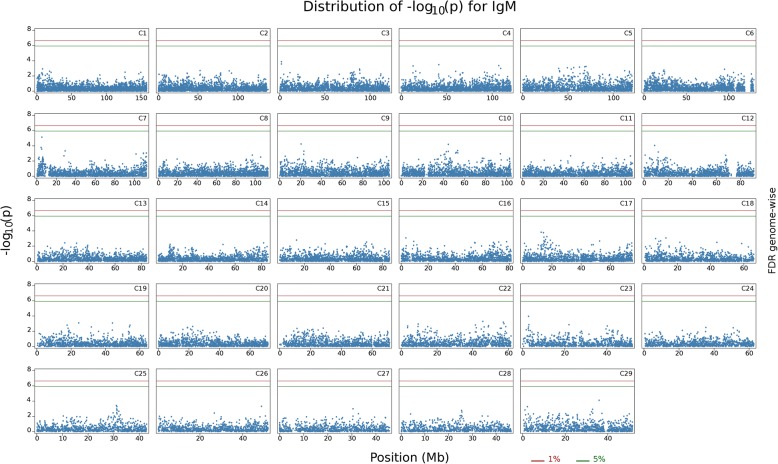
Distribution of -Log10 *P*-values from single SNP analyses for natural antibody isotype IgM binding KLH, for every chromosome. The red line indicates FDR rate of 1% and green line indicates FDR rate of 5%

**Fig. 2:**
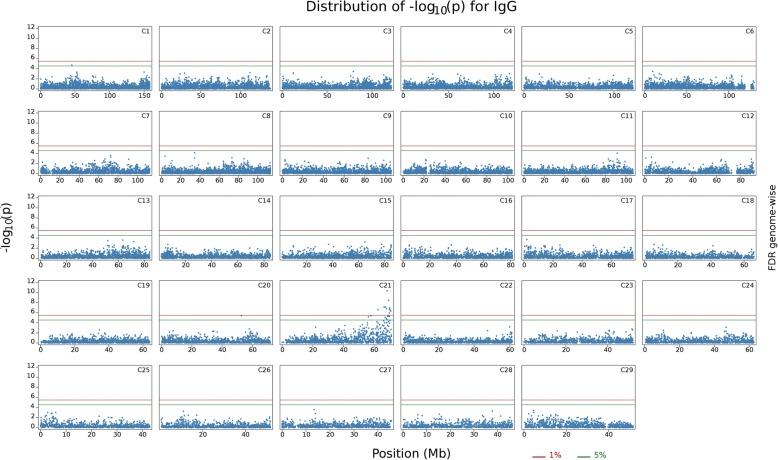
Distribution of -Log10 *P*-values from single SNP analyses for natural antibody isotype IgG binding KLH, for every chromosome. The red line indicates FDR rate of 1% and green line indicates FDR rate of 5%

**Fig. 3:**
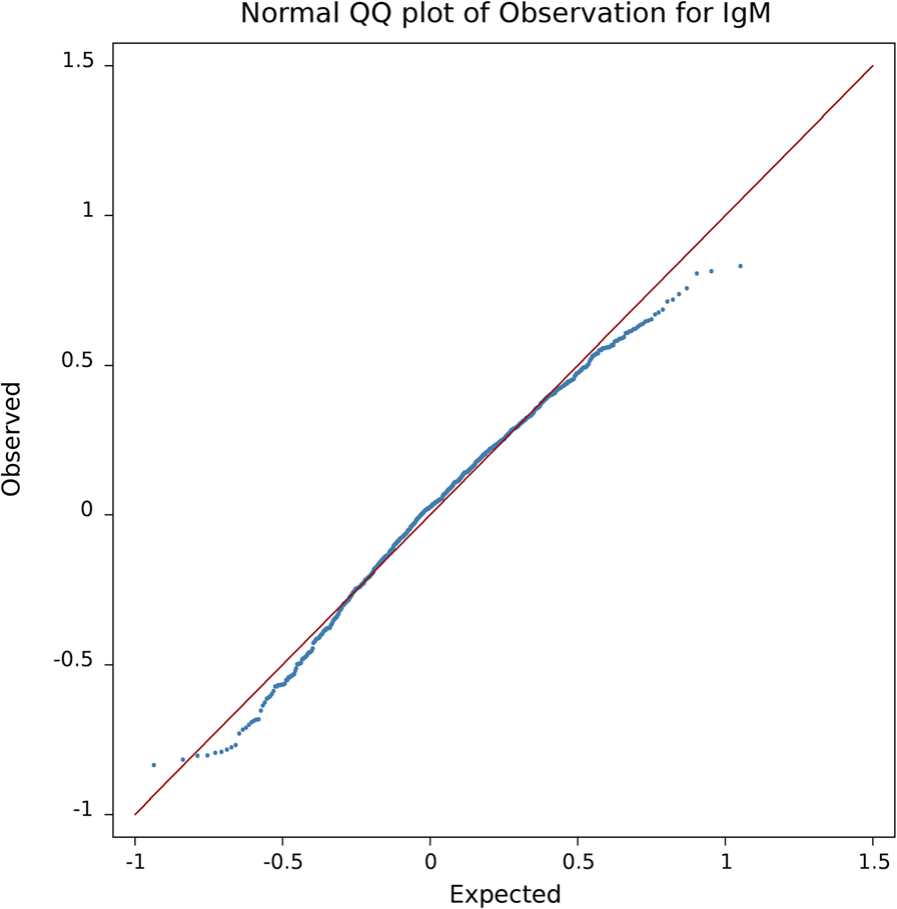
Q-Q plot of –Log10 *p*-values for natural antibody isotype IgM

**Fig. 4:**
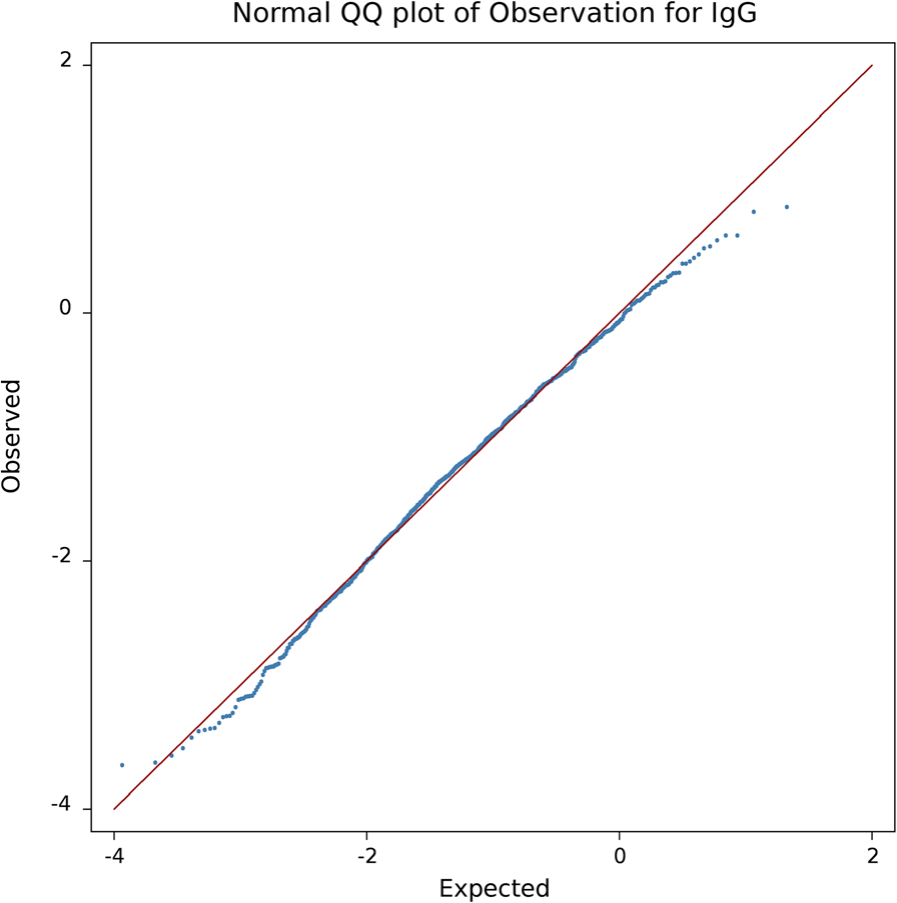
Q-Q plot of –Log10 p-values for natural antibody isotype IgG

At a genome-wide FDR of 5%, NAb IgM did not show any significant associations (Fig. 1 and Additional file 1: Figure S1). Significant associations between 23 SNPs and NAb IgG were found (Fig. 2, Additional file 2: Figure S2 and Table 2). The genomic inflation factor for IgG and IgM was 0.99 and .96, respectively. Figures 3 and 4 show Q-Q plots of test statistics. The significant SNPs were located on *Bos taurus* autosomal chromosome 1, (1 SNP), 20 (1 SNP) and 21 (21 SNPs). The region between the significant SNPs and the adjacent SNP on each side were screened to identify the positional candidate gene. The selected regions around each significant SNPs ranged from 51,008 bp to 276,066 bp with the average of 53,583 bp on each side of the significant SNPs. Thirty-seven genes were found on the bovine genome (UMD3.1, R.91) near the significant SNPs. Two of these genes were located on BTA-1 and the others were located on BTA-21 (Table 3). No gene was found around the significant SNP on BTA-20, despite the large region around this SNP (276,066 bp).

**Table 2:** The list of SNPs that are significantly associated with IgG NAb

No.	SNP ID	BTA:Position	MAF	ASE	FDR Corrected *p-value*
1	BTA-95285-NO-RS	1:45452593	0.4121	−0.3635	3.93E-02
2	ARS-BFGL-NGS-80731	20:52529599	0.4225	0.358795	1.15E-02
3	ARS-BFGL-NGS-36353	21:56272444	0.3745	0.373862	1.42E-02
4	HAPMAP36811-SCAFFOLD240339_791	21:57920570	0.0753	0.668197	1.16E-02
5	ARS-BFGL-NGS-18270	21:62445122	0.0966	0.669079	1.03E-03
6	ARS-BFGL-NGS-114799	21:66492068	0.4214	−0.44719	6.56E-04
7	ARS-BFGL-NGS-20339	21:67088847	0.3417	−0.37211	1.81E-02
8	ARS-BFGL-NGS-29558	21:67345260	0.2074	−0.52465	6.56E-04
9	ARS-BFGL-NGS-119656	21:67963614	0.1654	0.469889	3.17E-02
10	ARS-BFGL-NGS-21626	21:68105539	0.2091	−0.51097	9.35E-04
11	BTA-03263-RS29011028	21:68356479	0.2298	−0.39036	4.61E-02
12	ARS-BFGL-NGS-86477	21:68399787	0.3504	−0.54776	9.82E-07
13	HAPMAP41289-BTA-53093	21:68751694	0.0464	−0.82127	1.34E-02
14	ARS-BFGL-NGS-39941	21:69279283	0.2102	0.441591	1.25E-02
15	ARS-BFGL-NGS-115062	21:69395154	0.4973	−0.46877	5.27E-05
16	ARS-BFGL-NGS-109976	21:69891992	0.2959	0.422929	3.81E-03
17	ARS-BFGL-NGS-1345	21:69939350	0.4885	−0.36527	1.37E-02
18	ARS-BFGL-NGS-33371	21:69972343	0.4088	−0.56953	8.87E-08
19	ARS-BFGL-NGS-63968	21:70012413	0.0393	1.02312	9.35E-04
20	ARS-BFGL-NGS-10484	21:70050042	0.0486	0.926758	1.03E-03
21	ARS-BFGL-NGS-40895	21:70116946	0.4509	−0.41571	9.13E-04
22	ARS-BFGL-NGS-106472	21:70732448	0.0922	0.67538	1.74E-03
23	BTA-100472-NO-RS	21:70759096	0.185	−0.50167	1.49E-03

*BTA Bos taurus* autosome, *MAF* Minor Allele Frequency, *ASE* Allele Substitution Effect

**Table 3:** The list of positional candidate genes nearby SNPs that are significantly associated with IgG NAb

Gene stable ID	Gene name	Gene start (bp)	Chromosome Number
ENSBTAG00000012140	ADGRG7	45,415,066	1
**ENSBTAG00000016363**	**TFG**	**45,489,052**	**1**
ENSBTAG00000010416	RIN3	57,859,148	21
ENSBTAG00000018310	SETD3	66,064,391	21
ENSBTAG00000018636	CCNK	66,159,547	21
ENSBTAG00000025181	CCDC85C	66,175,707	21
**ENSBTAG00000013491**	**EML1**	**66,358,950**	**21**
ENSBTAG00000007233	WDR25	66,917,780	21
ENSBTAG00000017716	BEGAIN	67,072,575	21
ENSBTAG00000038073		68,149,406	21
ENSBTAG00000043578		68,150,604	21
ENSBTAG00000037977		68,151,114	21
ENSBTAG00000020192	PPP2R5C	68,377,171	21
ENSBTAG00000007007	WDR20	68,639,658	21
ENSBTAG00000007008	MOK	68,706,703	21
ENSBTAG00000007009		68,751,572	21
ENSBTAG00000044797	U6	68,761,089	21
ENSBTAG00000010993		68,778,065	21
ENSBTAG00000010995	CINP	68,789,514	21
ENSBTAG00000043100	U5	68,797,764	21
ENSBTAG00000012143	TECPR2	68,837,087	21
**ENSBTAG00000013475**	**TRAF3**	**69,228,047**	**21**
**ENSBTAG00000015750**	**AMN**	**69,266,381**	**21**
**ENSBTAG00000016456**	**CDC42BPB**	**69,279,222**	**21**
**ENSBTAG00000026893**	**EXOC3L4**	**69,418,979**	**21**
**ENSBTAG00000035995**	**TNFAIP2**	**69,455,152**	**21**
ENSBTAG00000021199	APOPT1	69,847,359	21
**ENSBTAG00000017299**	**KLC1**	**69,896,140**	**21**
**ENSBTAG00000009966**	**XRCC3**	**69,960,277**	**21**
ENSBTAG00000003556	ZFYVE21	69,978,023	21
ENSBTAG00000003559	PPP1R13B	69,982,291	21
ENSBTAG00000026886	MP68	70,113,045	21
ENSBTAG00000046186		70,114,200	21
ENSBTAG00000020402	TDRD9	70,123,917	21
ENSBTAG00000046401	RD3L	70,133,009	21
ENSBTAG00000022775	C21H14orf180	70,707,408	21
ENSBTAG00000006673	TMEM179	70,717,761	21

Candidate genes based on functional annotation analysis are shown in bold

The Over Representation Analysis (ORA) on the Gene Ontology (GO) terms associated with all the positional candidate genes (37 genes) revealed 3 significant over-represented terms (FDR *p*-value < 0.05): “thyroxine 5-deiodinase activity” (GO:0033798, for molecular function), “hormone biosynthetic process” (GO:0042446, for biological process) and “exocyst” (GO:0000145, for cellular compartment). It also should be noted that “exocytosis” (GO:0006887, for the biological process) was over-represented, but the FDR *p*-value was just above significant level (*p*-value = 0.076).

The functions of all 37 genes were individually checked to investigate any possible biological similarity between the candidate genes [28–30]. Seven genes (TFG on BTA-1, and EML1, AMN, CDC428BP, EXOC3L4, TNFAIP2, and KLC1 on BTA-21) were found among the candidate genes that are associated with intracellular transportation of vesicles. In addition, two genes (TRAF3 and XRCC3 on BTA-21) were found among the positional candidates which are associated with immunoglobulin class switching and somatic hypermutation of B-cells [31–34].

## Discussion

In this study no significant associations were found for NAb IgM even though the heritability of this trait was relatively high. This may indicate that the underlying genetic architecture of IgM is more polygenic with no outstanding major genes. For Isotype IgG significant associations were found on chromosome 1, 20 and 21 (BTA-1, 20 and 21). For both traits, several peaks across genomes were observed that were not significant. These suggestive peaks should be further investigated in a bigger sample size.

In order to check for existence of population stratification or family structure, the GWAS study was repeated without including the animal random effect (the results were not shown). Genomic inflation factors (λ) were 1.25 and 1.44 for IgG and IgM, respectively, showing severe inflation in *p*-values. After properly correcting for population stratification and family structure, λ should be close to 1 [35]. Incorporating the full genomic covariance structure between individuals by fitting the random additive effect in the model resulted in λ close to 1 for both traits (0.99 for IgG and 0.96 for IgM; Figs. 3 and 4).

The present study included NAb of isotypes IgG and IgM, binding KLH. Natural antibodies are considered as the humoral part of the innate immune system [6]. The model antigen KLH is assumed not to be present in the common daily environment of dairy cows, and therefore can be assumed as an antigen that cows are not exposed. Consequently, any antibody detected to KLH is likely NAb or possibly specific antibody cross reacting to antigens to which the individual has been exposed. These antigens may include self antigens and therefore caution must be exercised if selecting for increased amounts of NAb until any cross reacting self antigens can be determined. Generally, anti-KLH antibody is considered Nab [27].

Natural antibodies are thought to be an indicator of innate host defence. Moreover, NAb are variable between individual cows and are under substantial genetic control [11, 26, 27]. These findings, together with the ability of early protection and poly-reactivity of NAb, make them potential traits to aid in genetic selection for disease resistance.

In this GWAS, significant and suggestive associations between markers and NAb IgG were found on chromosome 1, 20 and 21 (BTA-1, 20 and 21). In this study to identify candidate regions, the immediate adjacent SNPs on each side of the significant SNPs were selected, instead of selecting a fixed size. In this approach, fewer genes can be identified compared to the fixed size approach, but it helps to remove noise by excluding genes when their adjacent SNPs are not significant.

The ORA showed “thyroxine 5-deiodinase activity” and “hormone biosynthetic process” as the most represented terms among the candidate genes. However, closer inspection revealed that both of these terms were overrepresented due to the presence of two genes (DIO3 and IDBG-647912) among the candidate genes. Both genes, along with a microRNA, are located in the vicinity of one SNP (21:68105539). Due to the lack of any supporting data that correlate the production of antibody with the function of these two genes, or the presence of genes with similar activity near other SNPs, this enrichment may be a type II error. However, it is possible that the microRNA (ENSBTAG00000038073) controls the production of these antibodies or there is one or more unknown genes in this region that control the production of antibody. Further investigation on the cellular compartment GO term (“exocyst”) revealed evidence to support the “trafficking of vesicles” as a candidate pathway. TFG, located on BTA-1, is responsible for the trafficking of newly synthesized protein from the endoplasmic reticulum (ER) to golgi apparatus (GA). KLC1, located on BTA-21, is responsible for retro-trafficking of vesicles from GA to ER; and AMN, on BTA-21, is responsible for the trafficking of vesicles from GA to the plasma membrane (PM). In addition, EXOC3L4 and TNFAIP2, are responsible in exocytosis, the process of releasing vesicle content to the extracellular environment. Moreover, EML1 and CDC42BPB play a role in organization of cytoskeleton that provides a pathway for the movement of vesicles inside cells in the process of exocytosis [36]. The molecular pathway of Ab production by B lymphocytes, involves several glycosylation steps that requires trafficking between ER, GA, and PM and the final release of antibody from the cell [37]. This pathway aligns with the function of these seven candidate genes. Moreover, the presence of TRAF3, with a major role in class-switching of immunoglobulin, and XRCC3, with a role in DNA recombination and hypermutation in B cells, align with the isotype of the NAb that was measured in in this study [31–34].

These results from the pathway analysis also point out the similarity between the production of SpAb and NAb. It has been previously shown that B1 cells in mice producing IgG and IgA NAb undergo some level of affinity maturation [38, 39]. Given the fact that affinity maturation happens after the presentation of antigens to the B cells, it is possible that B1 cells are also exposed to an antigen to produce IgG. The effect of antigen on B1 lymphocytes and NAb has been shown by the reduced level of IgG NAb, but not IgM, in germ-free antigen-free mice [40], however, the source of the antigenic signal has not yet been found. T cell-independent antigens from microbiota and self-antigens were proposed as the source of antigenic stimulation [38].

Recently, the composition of gut microbiota was found to be correlated with the level of poly-reactive IgA in germ-free mice, but this correlation was caused by the genetic structure of the mice rather than gut microbiota [41]. It is noteworthy to mention that genetic association studies have not found any significant correlation between the SpAb and NAb. The genetic correlation in different studies can be found when the phenotype is based on NAb even against different antigens or isotypes rather than between SpAb and NAb [11, 25–27, 42].

The available information from GWAS of NAb in cows is limited, but BTA-21 is in common between this study and the study by Wijga et al. [43]. However, there was no similarity between markers associated with SpAb and NAb. From the immunological perspective, it can be hypothesized that the main signals from antigen presenting cells to B2 lymphocytes probably overcome the effect of vesicle trafficking at the level of Ab production, but in the absence of strong classical signal, vesicle trafficking gains more effect on the production of NAb.

From the breeding perspective, the absence of correlation between NAb and SpAb provides an opportunity to include NAb as a new trait to improve dairy cattle health given the notable heritability of NAb. Despite numerous reports on the beneficial role of IgM NAb and to a lesser extent IgG NAb in mice [38, 39, 44], the beneficial correlations reported between NAb and resistance against infectious diseases are minimal in livestock [27, 45, 46]. Therefore, the role of NAb in immunity of livestock needs further investigation. It should also be noted that a number of studies are proposing self-antigens as the stimulator of B1 cells and reporting both negative and positive roles of self-reactive NAb in autoimmune diseases [39]. In mice, lupus-like autoimmune diseases developed when NAb production was impaired, but mice that could produce only IgM NAb, but not IgG NAb, did not develop the autoimmune disease [47, 48]. Therefore, to develop breeding strategies to increase NAb in animals with relatively long lifespan such as cattle the isotype of NAb and antigen should be cautiously explored and animals monitored for any signs of autoimmunity.

## Conclusion

The level of NAb in cattle is moderately heritable. Despite similar heritability of IgG and IgM classes of NAb, the structure of genes that are associated with them seem to be different. The absence of any significant SNPs with IgM, given the sample size of this study, is likely representing the polygenetic control of the production of IgM. In the case of IgG, BTA-1,20 and 21 seem to carry the main quantitative trait loci. Based on the function of positional candidate genes that were found in this study, vesicle trafficking in B1 cells from production to secretion of IgG NAb play an important role on the level of IgG NAb in serum. Self-reactive antigens have been reported as stimulators of B1-NAb-producing cells and therefore caution must be exercised if selection based on NAb is to be developed.

## Methods

### Animals and phenotypes

Blood serum samples were taken of 1327 cows from 64 privately owned herds in Canada. Cows were on average 161 days in lactation when the blood samples were taken, with a range from 0 to 498, and 13 cows with DIM > 500. Cows had on average a parity of 2.16, ranging from 0 to 12. Number of sampled cows per farm ranged from 2 to 160 cows per farm. The cows were the offspring of 472 unique bulls (on average: 2.77 cows per sire) and 1010 unique dams. The pedigree contained 13,128 animals and was provided by the Canadian Dairy Network (CDN; Guelph, Ontario, Canada). Immune response phenotypes used in this study were based on NAb of the isotypes, IgG and IgM, tested against the model antigen, not previously seen by cattle, keyhole limpet hemocyanin (KLH). To obtain the NAb phenotypes an indirect ELISA procedure was used, as described by Thompson-Crispi (2013a). Briefly, 96 well plates were coated with 5 μg/ml KLH) (MP Biomedicals, Solon, OH) in carbonate-bicarbonate buffer (pH 9.6), and incubated over night at a temperature of 4 °C. Next day, plates were washed 3 times with PBS and 0.05% Tween 20 (Sigma-Aldrich Canada Ltd., Oakville, ON, Canada) (wash buffer pH 7.4) and blocked with PBS, 3% Tween 20, 1,5% BSA and 1.5% FCS for 1 h at room temperature (RT) then washed again 3 times. Four serial dilutions starting with 1/40 of the serum samples in wash buffer were added to the plate and incubated for 2 h at RT. Plates were wash 5 times and the secondary antibodies conjugated to alkaline phosphatase dissolved in Tris-Tween buffer with 0.05% Tween 20 (pH 7.4) were added to the plates: either 1: 10.000 monoclonal anti-bovine IgG from mouse ascites fluid (Sigma-Aldrich, St. Louis. MO, USA) or 1:5000 anti-bovine IgM produced in sheep (Bethyl Laboratories, Montgomery, TX, USA) and incubated for 1 h at RT. All wash steps were performed with ELx405 Auto Plate washer (Biotek Instruments Inc., Winooski, USA). Substrate (p-nitrophenyl phosphate) (Sigma-Aldrich Canada Ltd., Oakville, On, Canada) was added and incubated for about 30–60 min. Optical density (OD) values at 405 nm were obtained using EL808 plate reader (BioTek Instruments Inc., Winooski, VT, USA). Optical density values were corrected to the rolling mean of the positive controls for each plate to account for day and plate variation, as described by Heriazon et al. [49]. The dilutions of the corrected OD values were summed and duplicates averaged for statistical analysis. NAb, both IgG and IgM isotype, were log-transformed to accomplish normality. After removing outliers for both IgG and IgM by Median Absolute Deviation method (considering three equivalent standard deviations). All experimental procedures were approved by the Animal Care Committee of the University of Guelph under guidelines of the Canadian Council of Animal Care.

### Genotyping and quality control

DNA was extracted from hair follicles and genotyping was performed with the Illumina Bovine SNP50 BeadChip by Zoetis Canada (Kirkland, Quebec, Canada). The initial dataset contained 45,187 SNP markers that are used in routine official genomic evaluation in Canada by the Canadian Dairy Network (CDN) (Guelph, ON, Canada). Details of quality control were explained in Wiggans et al. [50]. In the present study SNPs located on the X chromosome were not included and due to relatively small sample size, SNPs with MAF < 1% in the 1716 SNPs were excluded resulting in 43,471 SNPs for GWAS. The sporadic missing genotypes were imputed using 50,000 reference Holsteins from the CDN database by FImpute software [51]. After considering all the quality control measures, 925 genotyped cows were included in the association study.

### Statistical analysis

The association of the individual NAb with each individual SNP was estimated following a univariate mixed linear model:

$$ \mathrm{y}=\mathrm{Xb}+\mathrm{c}\beta +\mathrm{Wg}+\mathrm{e} $$

where y is the log-transformed corrected OD for NAb, b is a vector of fixed effects including overall mean, days in milk (classes: 1 = 0-20dim, 2 = 21–105 dim, 3 = 106-235dim, 4 > 235dim), parity (classes: 0 = heifers before calving, 1 = parity 1, 2 = parity 2, 3 = parity 3 and 4 = parity 4 and higher), herd (classes: 1 to 64), *β* is the gene substitution effect for the SNP, g is the random genetic effects, e is the random residual effects, X is an incidence matrix relating elements of b to y, c is a vector of genotypes for the SNP coded as 0 = BB, 1 = AB, 2 = AA and W is a standardized genotype matrix with element ${\mathrm{w}}_{ij}=\left({c}_{ij}-2{p}_i\right)/\sqrt{2\sum p\left(1-p\right)}$, where *p*_*i*_ is the allele frequency of the *i*th SNP and *c*_*ij*_ is genotype of *i*th SNP of *j*th individual.

The co-variance matrix for the vector y is:

$$ \mathrm{V}=\mathrm{G}{\sigma}_g^2+\mathrm{I}{\sigma}_e^2 $$

with $\mathrm{g}\sim N\left(0,\mathrm{G}{\sigma}_g^2\right)$ and$\mathrm{e}\sim N\left(0,\mathrm{I}{\sigma}_e^2\right)$, where ${\sigma}_g^2$ and ${\sigma}_e^2$ denote variance of random genetic effects and residual variance, respectively. G is the genomic relationship matrix calculated according to VanRaden (2008) using genome-wide SNP information as G = WW^′^ [52].

Additive genetic variance, residual variance and subsequently heritability were estimated with restricted maximum likelihood (REML) method and the average information algorithm [53].

Fitting random animal effect with the use of genomic relationship matrix prevents false-positives association due to population stratification and cryptic relationships between individuals and also increases the power [54]. Therefore, the above model should be proper for the Holstein population that has strong family structure due to the widespread use of few top bulls each year.

Inflation or deflation in *p*-values due to stratification or family structure was assessed by genomic inflation factor (λ) and also visually inspected by quantile-quantile (Q-Q) plot. λ is calculated as the median of the χ^2^ test statistics (1 degree of freedom) divided by its theoretical median under the null distribution [35]. In order to adjust for multiple comparisons, false discovery rate was controlled at 1 and 5% genome-wise levels [55]. GWAS and heritability estimation were carried out by snp1101 software [51].

### Functional annotation of the positional candidate genes

To identify the positional candidate genes that are associated with NAb, the genomic regions around the significant SNPs (FDR corrected *p*-value < 0.05) up to the next immediate SNP in both directions (before and after the significant SNP) were selected. These regions were cross-referenced against the cow genome (UMD 3.1, Ensemble Genes Release 91) using the BioMart tool in the Ensemble website to identify genes that are located in the vicinity of the significant SNPs. The extracted genes were then submitted into the innateDB online database to identify the GO terms that are associated with the candidate genes and subsequent ORA [30, 56].

## Additional files

Additional file 1: **Figure S1.** Distribution of -Log10 *P*-values from single SNP analyses for natural antibody isotype IgM binding KLH for each chromosome, separately. The red line indicates FDR rate of 1% and green line indicates FDR rate of 5%. (PDF 3430 kb)

Additional file 2: **Figure S2.** Distribution of -Log10 *P*-values from single SNP analyses for natural antibody isotype IgG binding KLH for each chromosome, separately. The red line indicates FDR rate of 1% and green line indicates FDR rate of 5%. (PDF 3180 kb)

## Abbreviations

Ab: Antibody
AMIR: Antibody-mediated immune response
BTA: *Bos taurus* autosomal chromosome
CMIR: Cell-mediated immune response
FDR: False discovery rate
GO: Gene ontology
GWAS: Genome-wide association study
Ig: Immunoglobulin
KLH: Keyhole limpet hemocyanin
LPS: Lipopolysaccharides
NAb: Natural antibodies
ORA: Over Representation Analysis
SCC or SCS: Somatic cell count or score
SNP: Single nucleotide polymorphism
SpAb: Specific antibodies
