# Supplementary material for: A genome-wide association study for natural antibodies measured in blood of Canadian Holstein cows
Source: BMC Genomics. 2018 Sep 21;19:694. doi: 10.1186/s12864-018-5062-6 (PMC6150957; doi:10.1186/s12864-018-5062-6)
Supplement: Supplementary file 2 — Figure S2. Distribution of -Log10 P-values from single SNP analyses for natural antibody isotype IgG binding KLH for each chromosome, separately. The red line indicates FDR rate of 1% and green line indicates FDR rate of 5%. (PDF 3180 kb) [file 12864_2018_5062_MOESM2_ESM.pdf]

Distribution of -log$_{10}$(p) for IgG

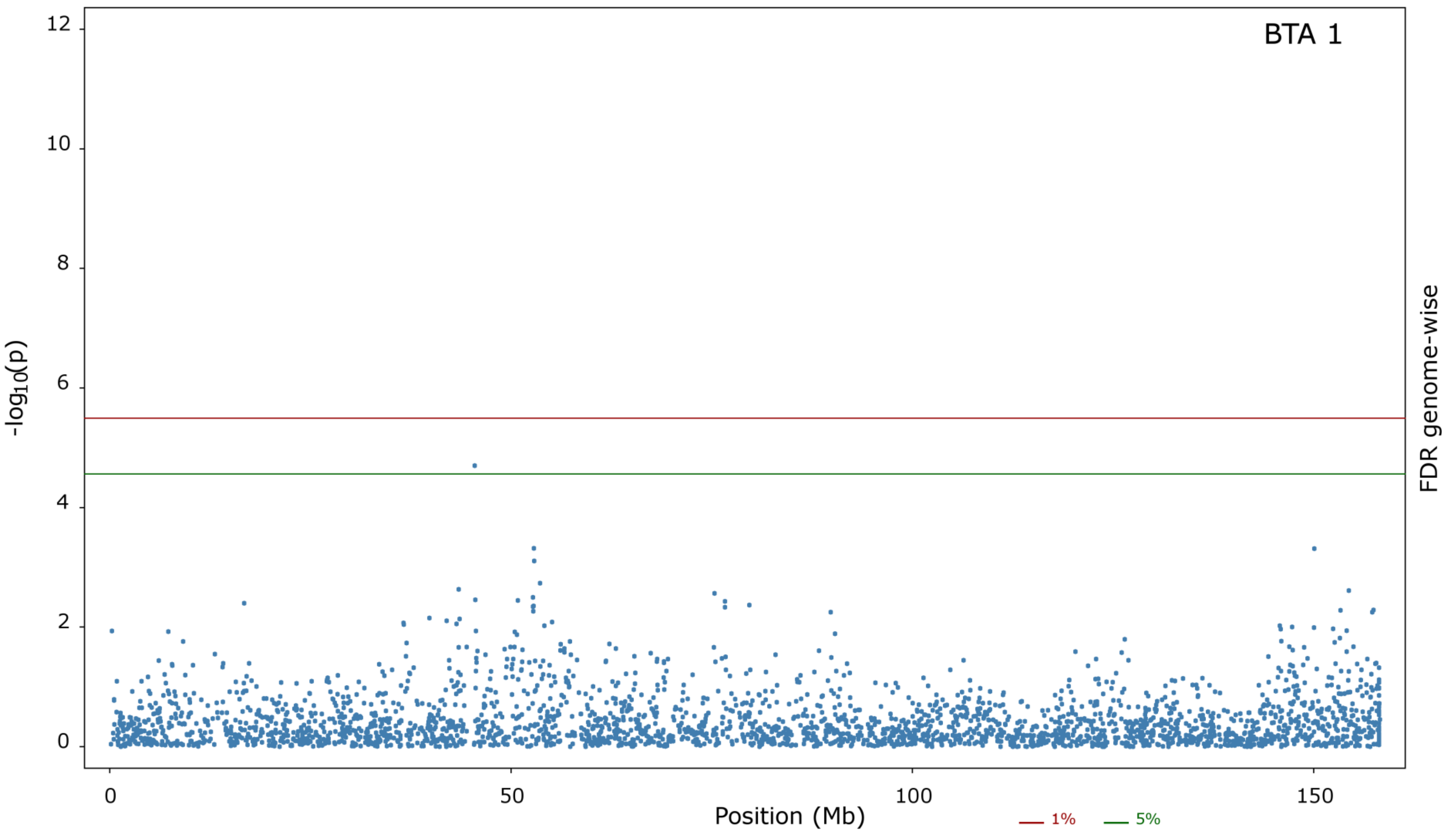

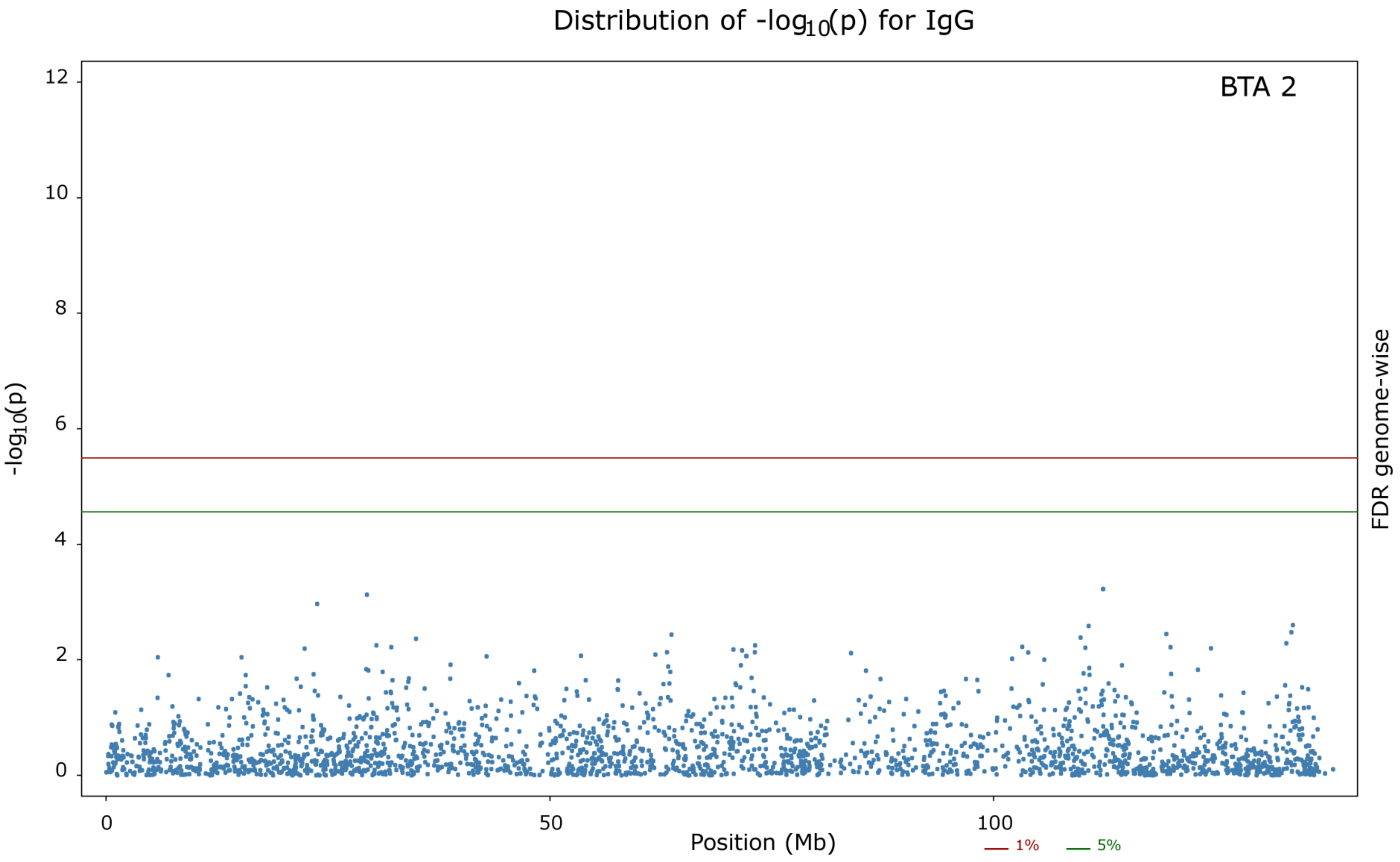

Distribution of -log10(p) for IgG
BTA 2
-log10(p)
FDR genome-wise
Position (Mb)
0
2
4
6
8
10
12
50
100
1%
5%

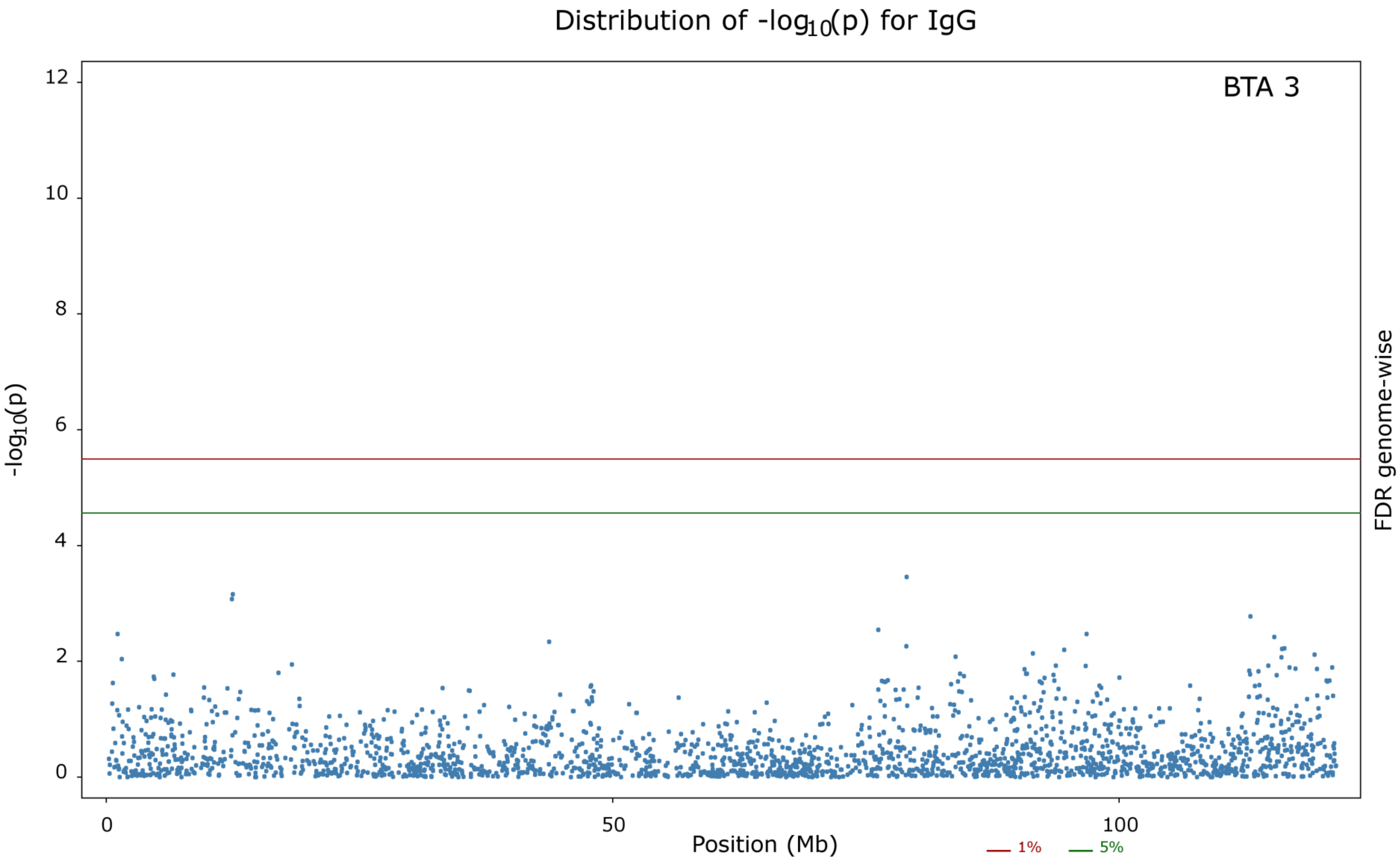

Distribution of -log10(p) for IgG
BTA 3
-log10(p)
12
10
8
6
4
2
0
FDR genome-wise
0
50
100
Position (Mb)
1%
5%

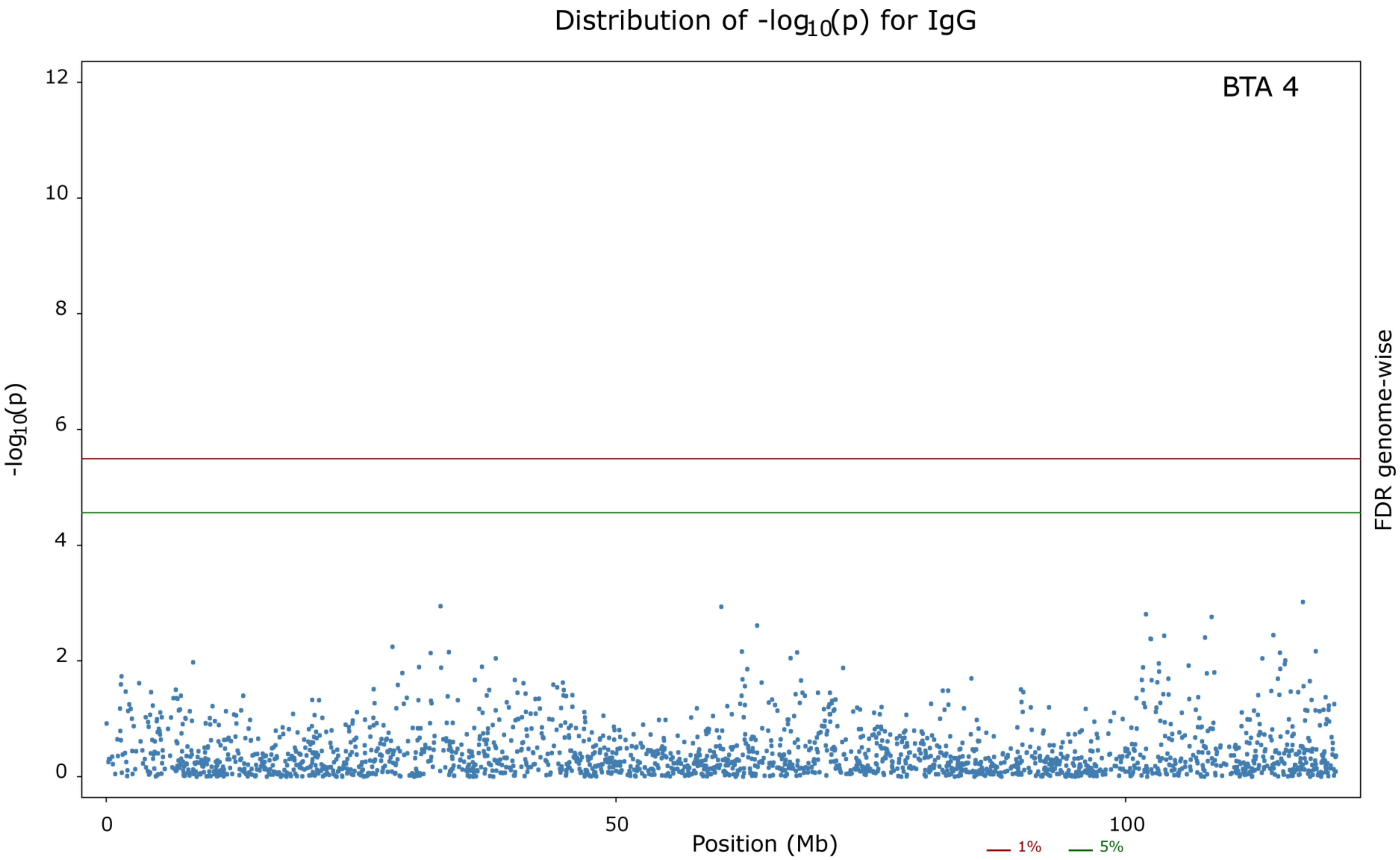

Distribution of -log10(p) for IgG
BTA 4
-log10(p)
FDR genome-wise
Position (Mb)
1%
5%
0
50
100
0
2
4
6
8
10
12

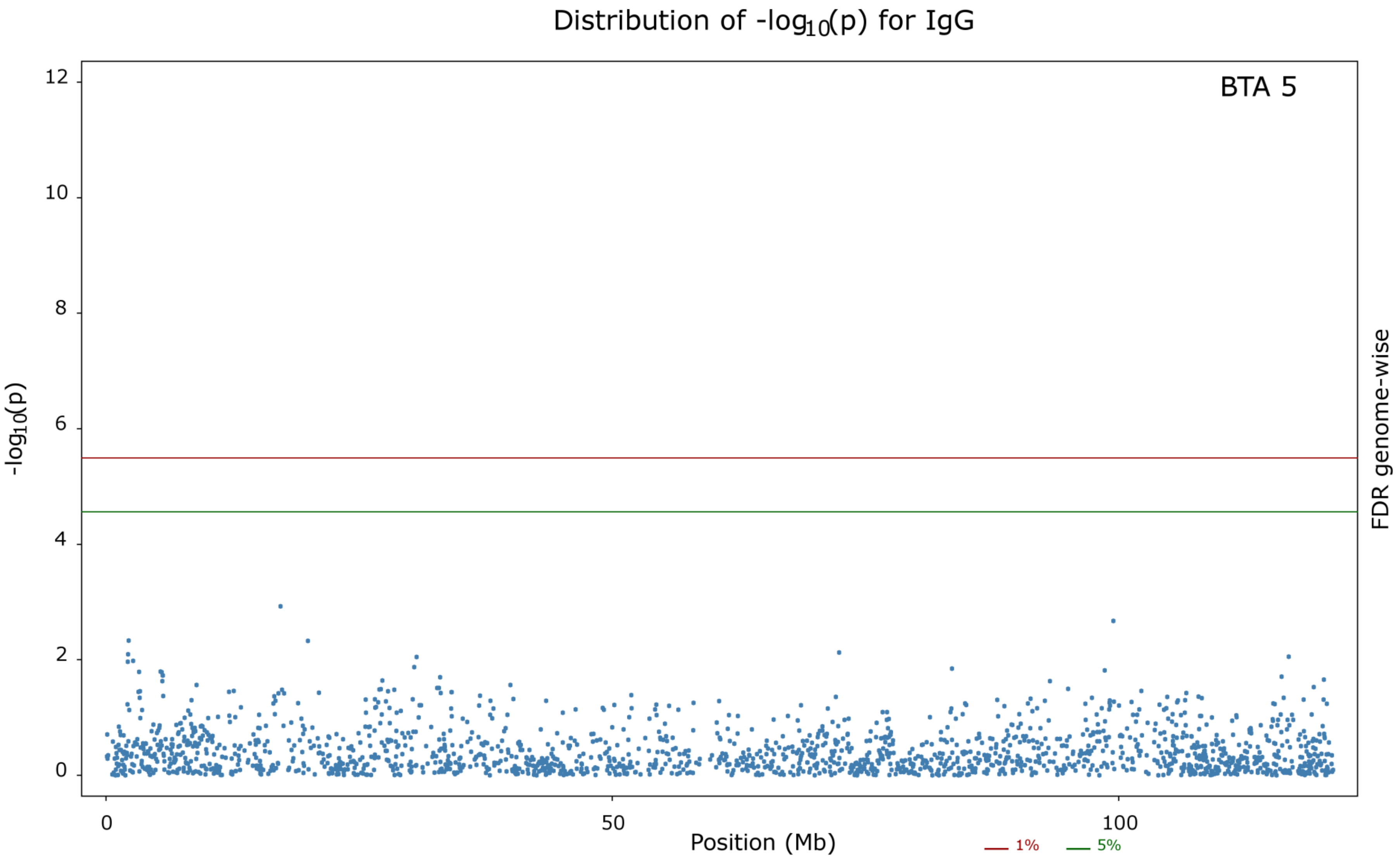

Distribution of -log10(p) for IgG
BTA 5
-log10(p)
12
10
8
6
4
2
0
0
50
100
Position (Mb)
1%
5%
FDR genome-wise

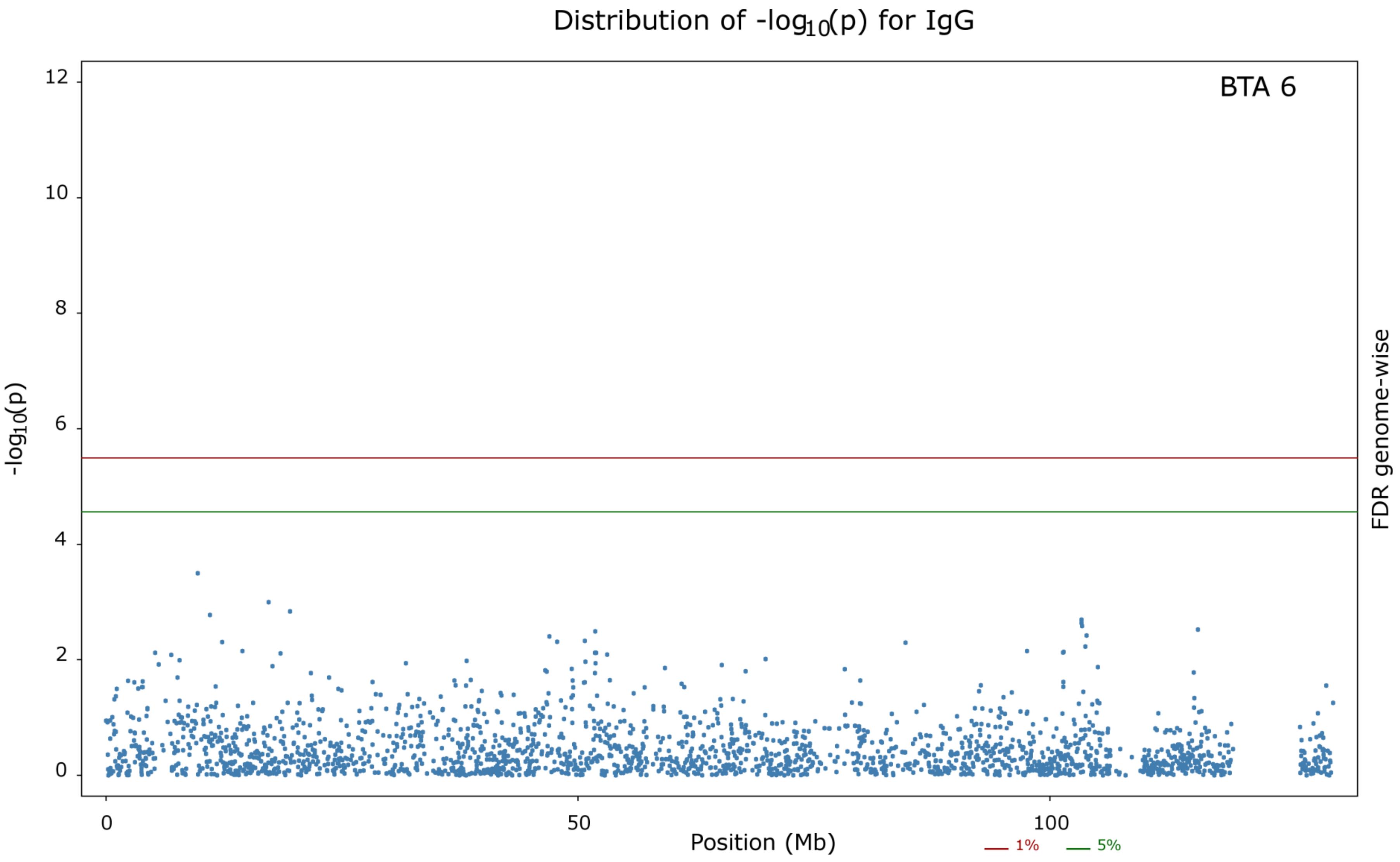

Distribution of -log10(p) for IgG
BTA 6
-log10(p)
12
10
8
6
4
2
0
50
100
Position (Mb)
1%
5%
FDR genome-wise

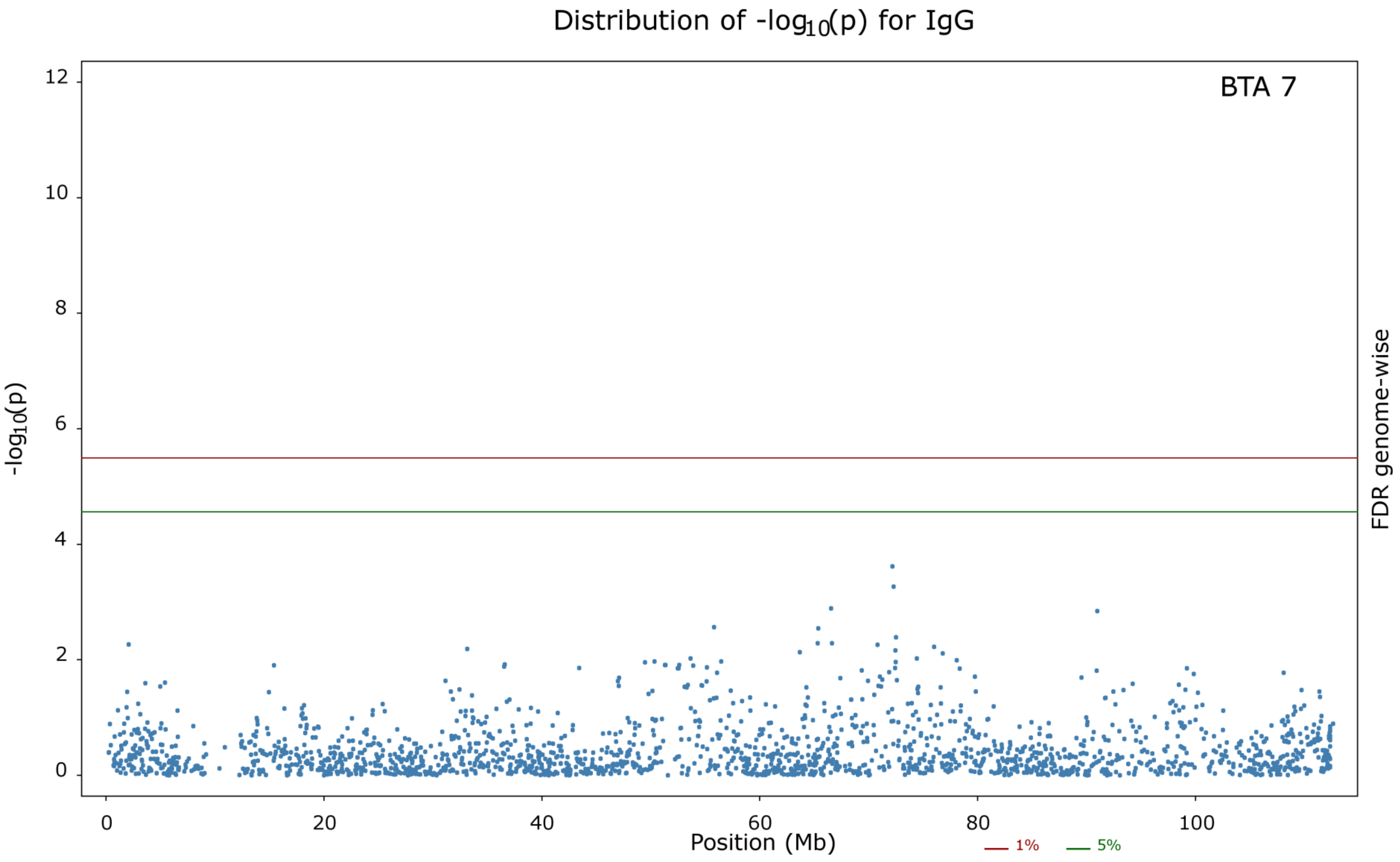

Distribution of -log10(p) for IgG
BTA 7
-log10(p)
0
2
4
6
8
10
12
0
20
40
60
80
100
Position (Mb)
1%
5%
FDR genome-wise

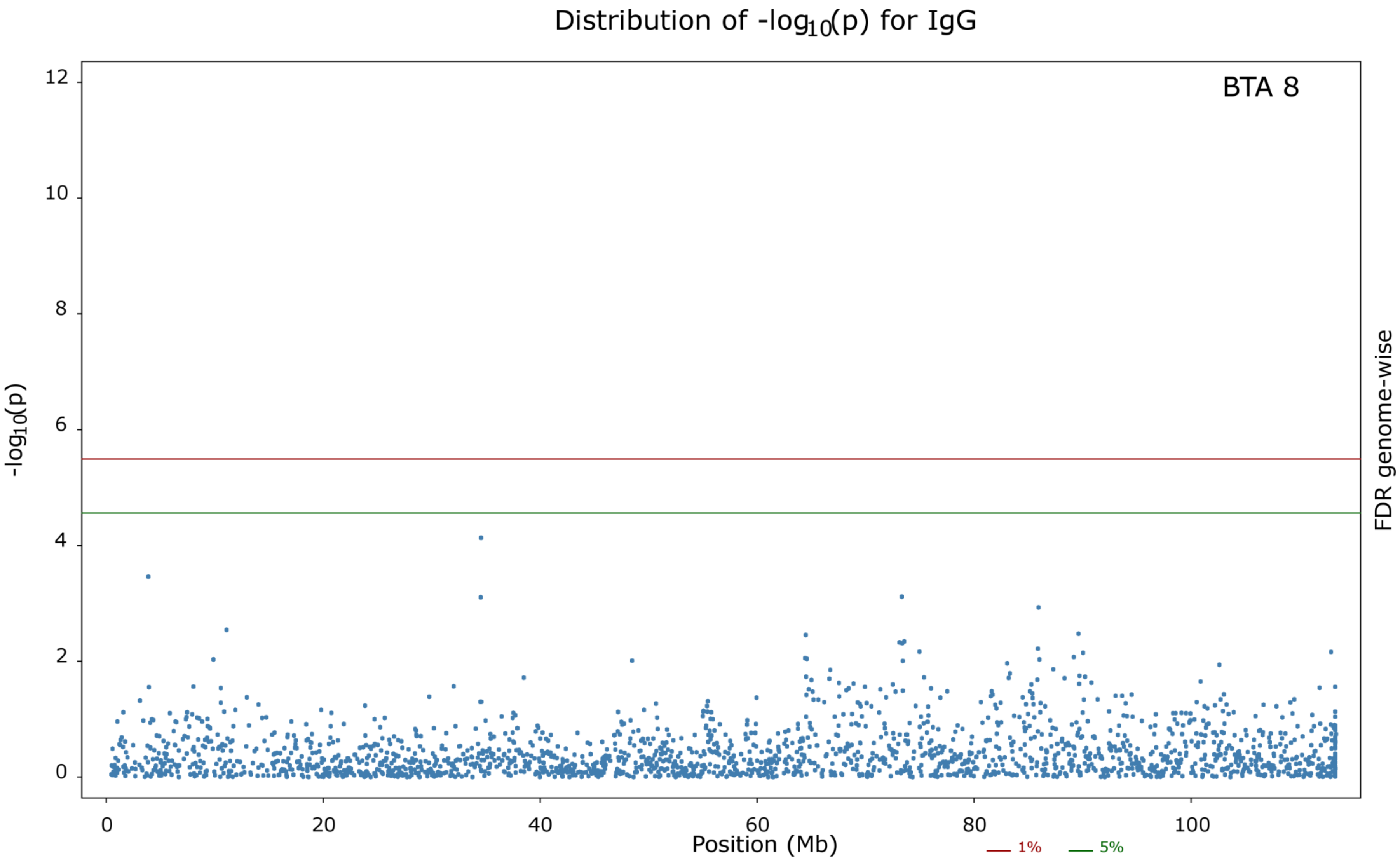

Distribution of -log10(p) for IgG
BTA 8
-log10(p)
Position (Mb)
FDR genome-wise
1%
5%
0
2
4
6
8
10
12
0
20
40
60
80
100

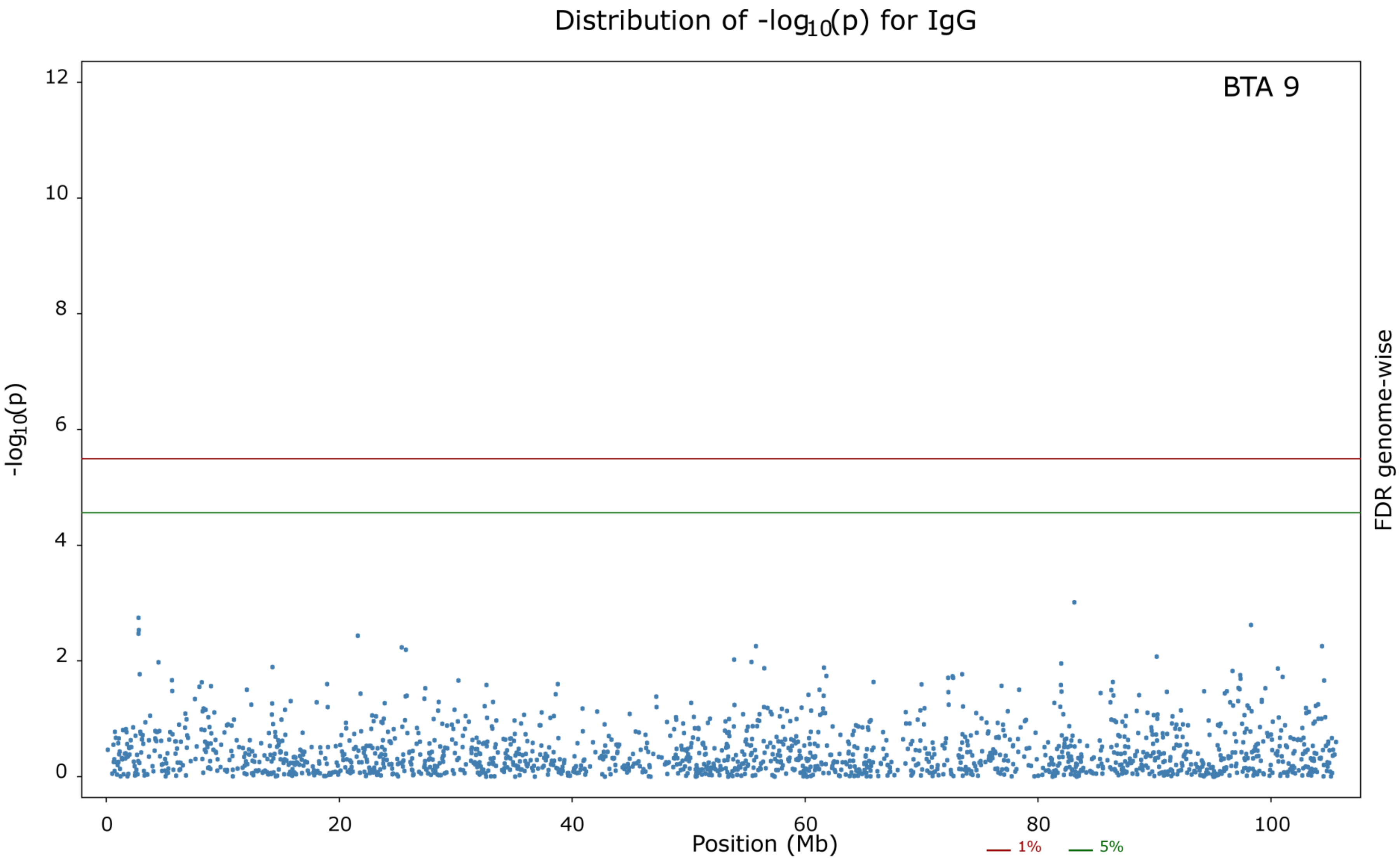
Distribution of -log10(p) for IgG
BTA 9
-log10(p)
12
10
8
6
4
2
0
20
40
60
80
100
Position (Mb)
FDR genome-wise
1%
5%

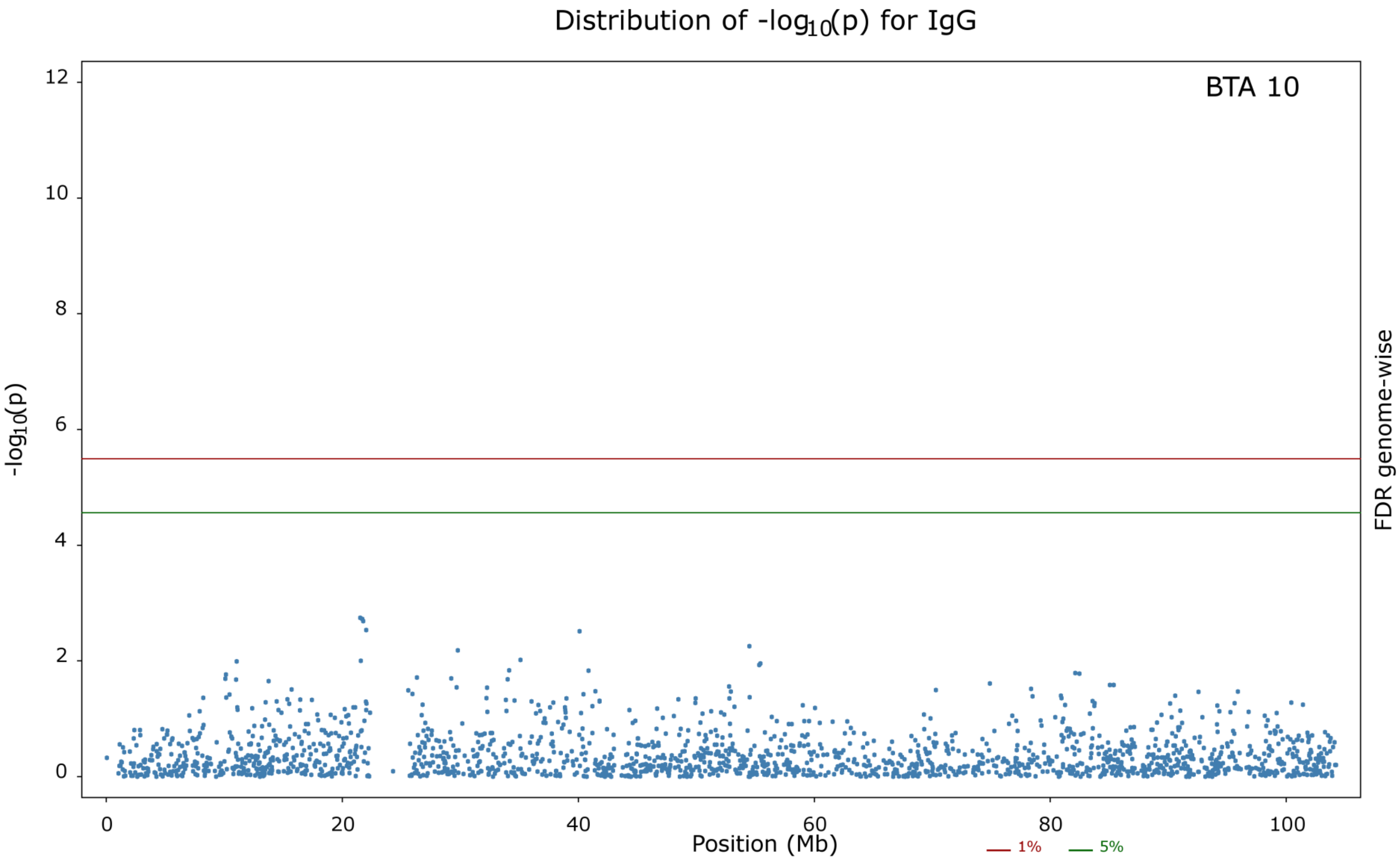
Distribution of -log10(p) for IgG
BTA 10
-log10(p)
FDR genome-wise
Position (Mb)
0
2
4
6
8
10
12
0
20
40
60
80
100
1%
5%

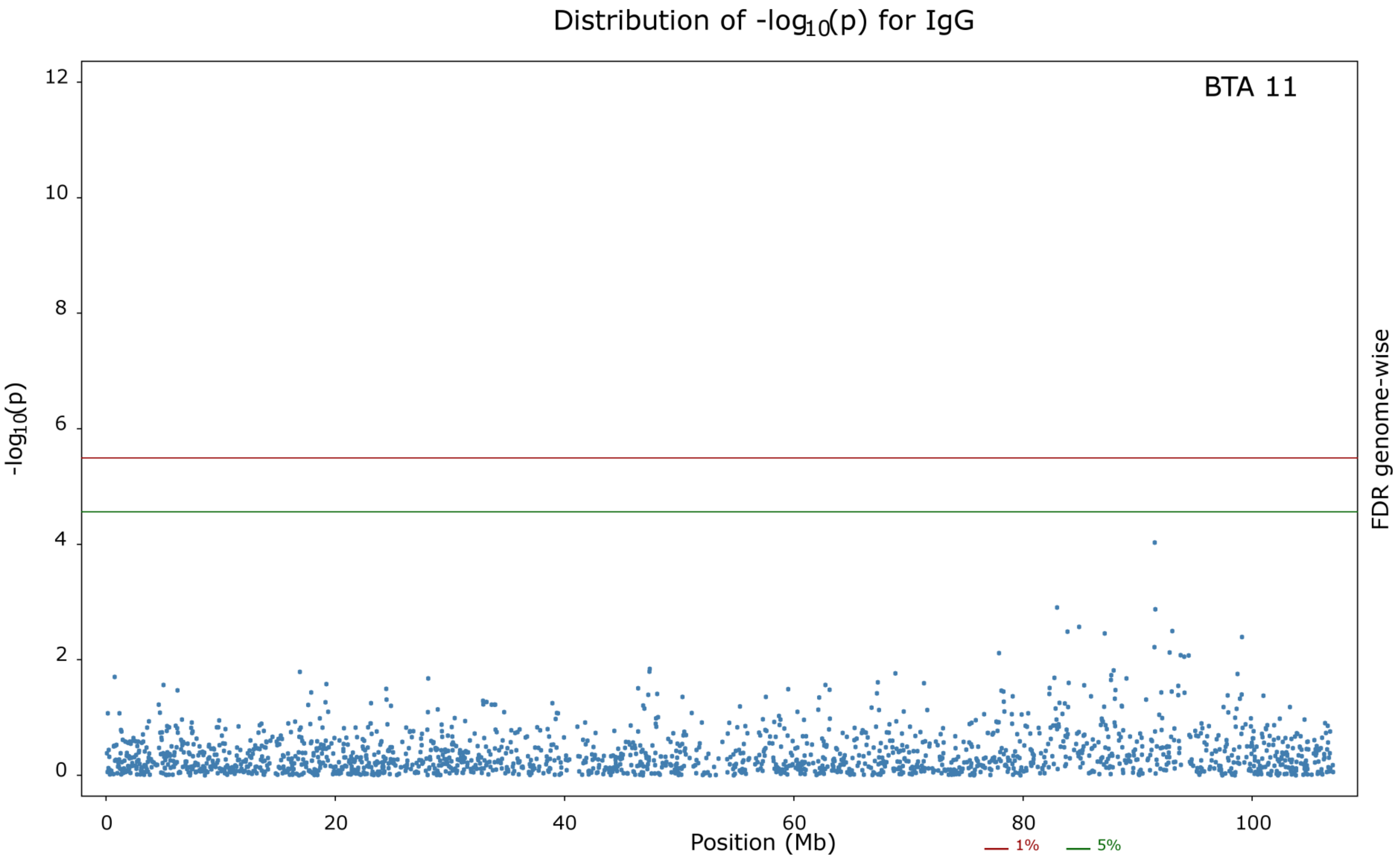

Distribution of -log10(p) for IgG
BTA 11
-log10(p)
FDR genome-wise
Position (Mb)
1%
5%
0
2
4
6
8
10
12
0
20
40
60
80
100

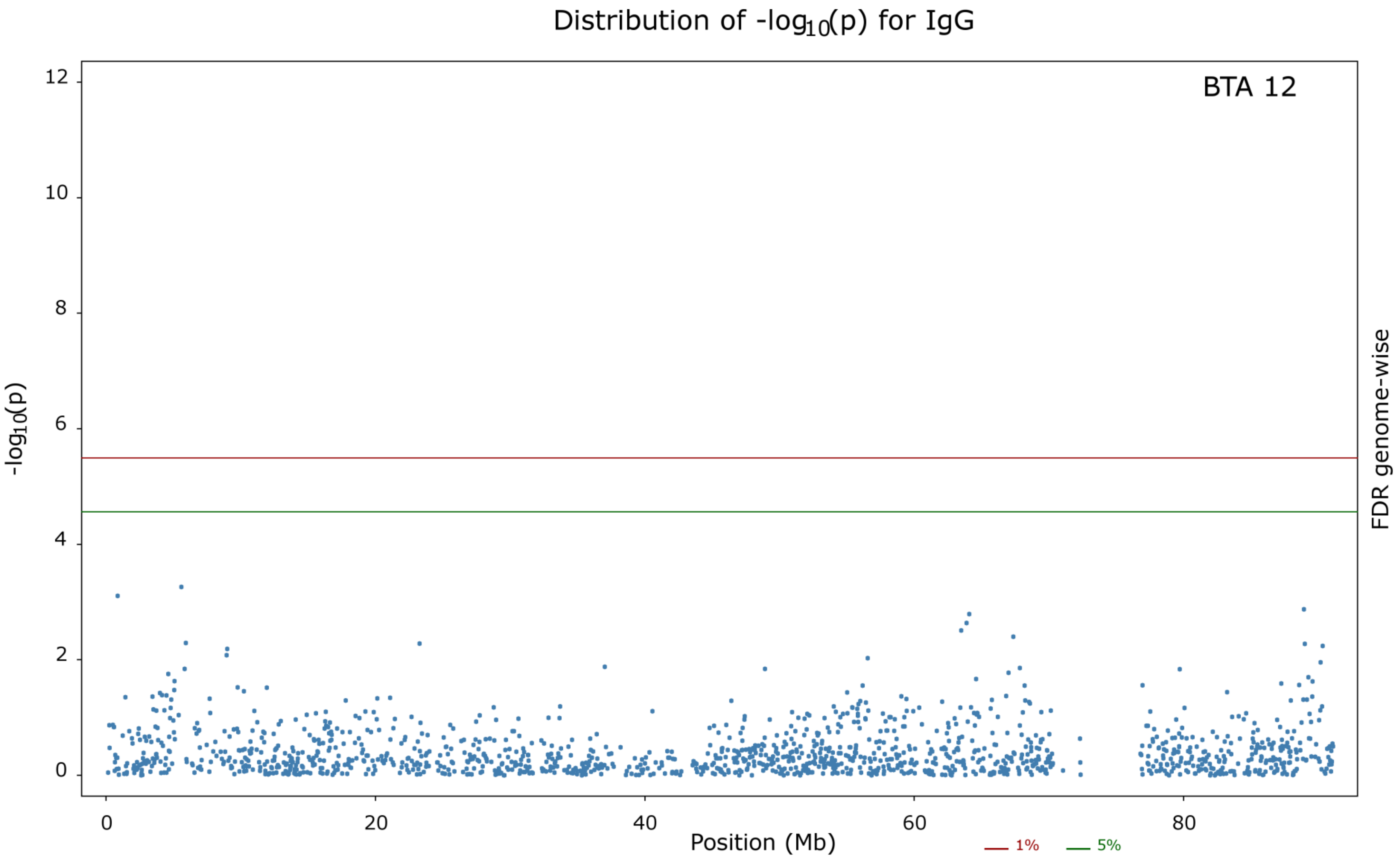

Distribution of -log10(p) for IgG
BTA 12
-log10(p)
0
2
4
6
8
10
12
0
20
40
60
80
Position (Mb)
FDR genome-wise
1%
5%

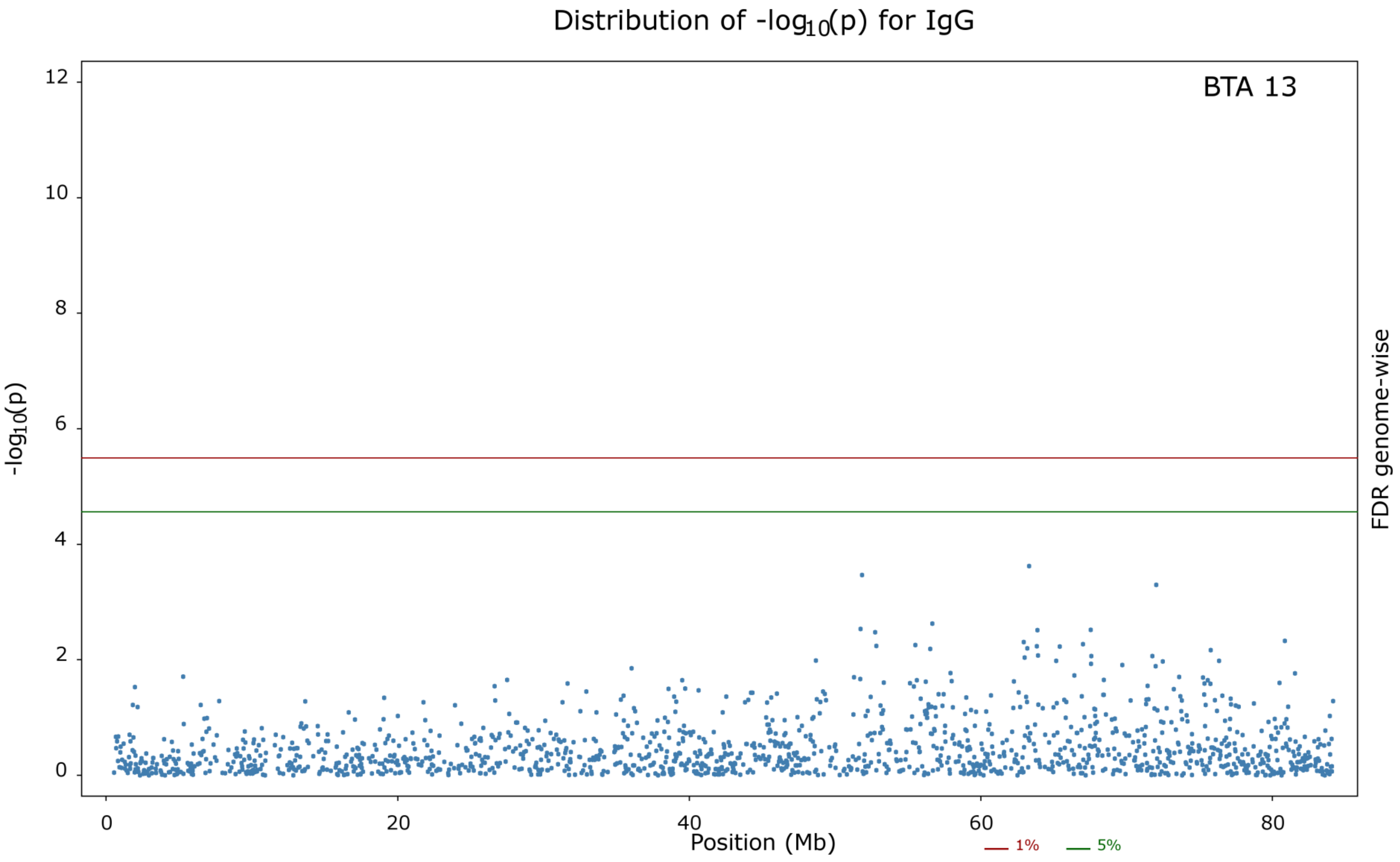

Distribution of -log10(p) for IgG
BTA 13
-log10(p)
FDR genome-wise
Position (Mb)
1%
5%
0
2
4
6
8
10
12
0
20
40
60
80

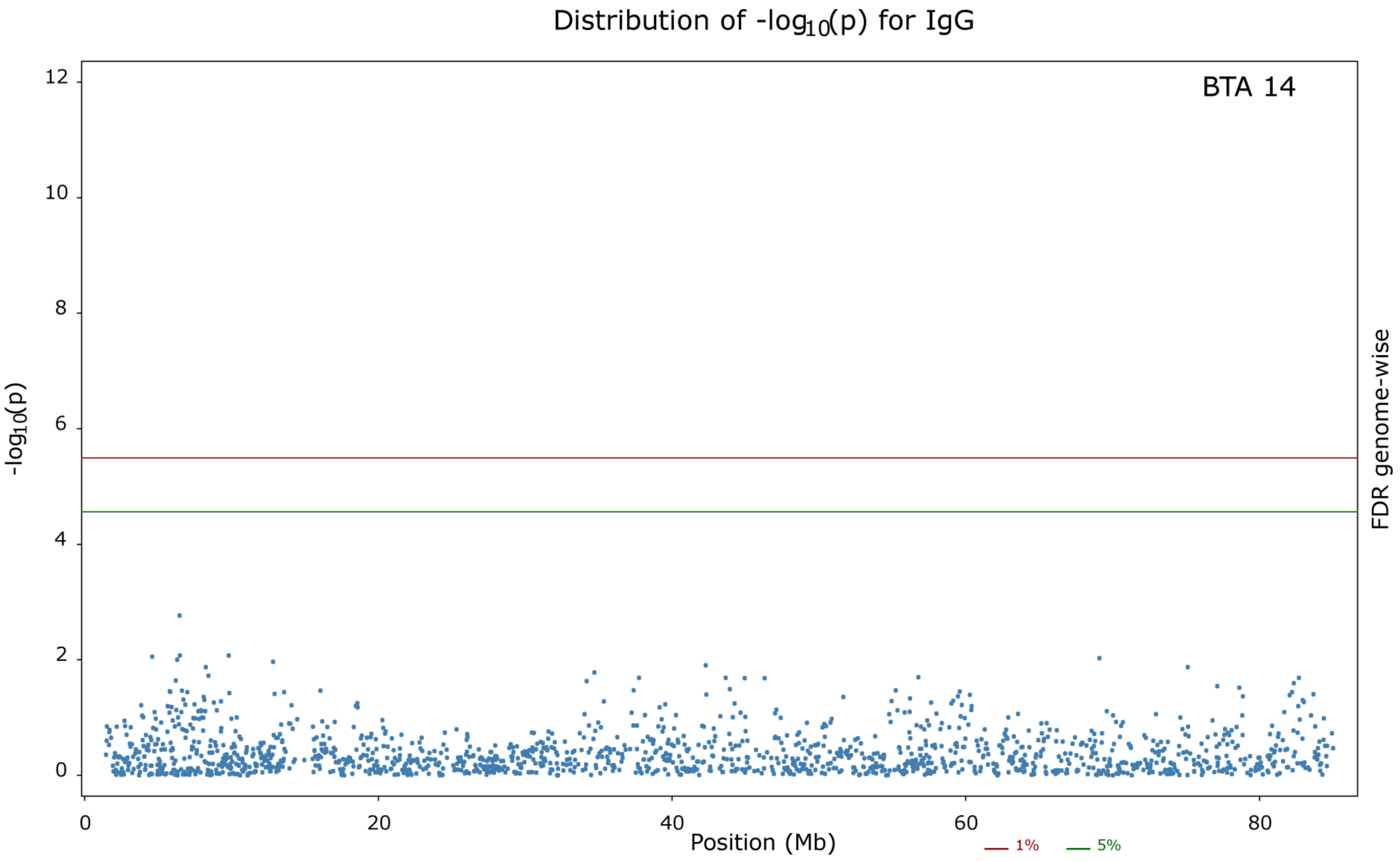
Distribution of -log10(p) for IgG
BTA 14
-log10(p)
FDR genome-wise
Position (Mb)
1%
5%
0
2
4
6
8
10
12
0
20
40
60
80

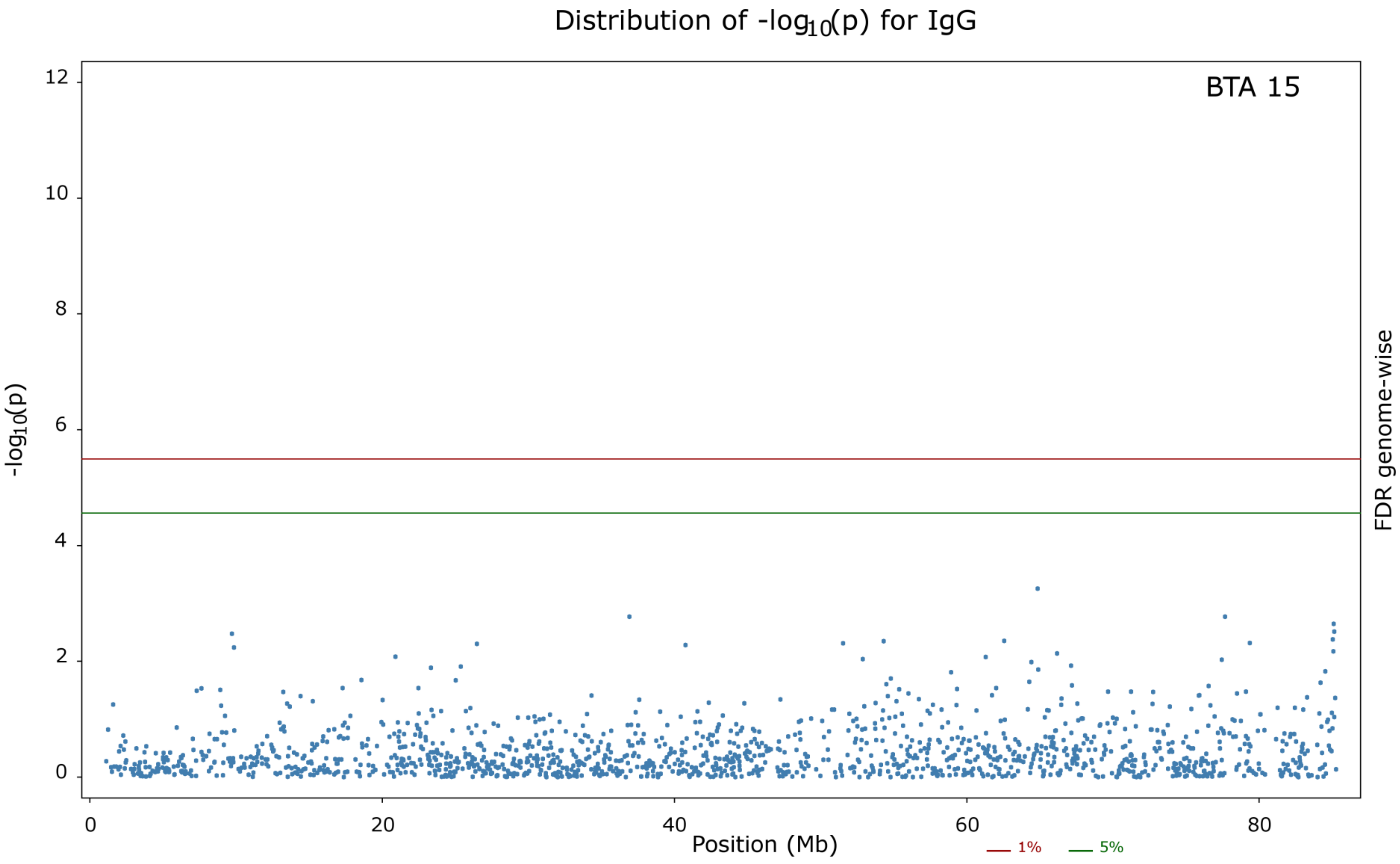

Distribution of -log10(p) for IgG
BTA 15
-log10(p)
12
10
8
6
4
2
0
0
20
40
60
80
Position (Mb)
FDR genome-wise
1%
5%

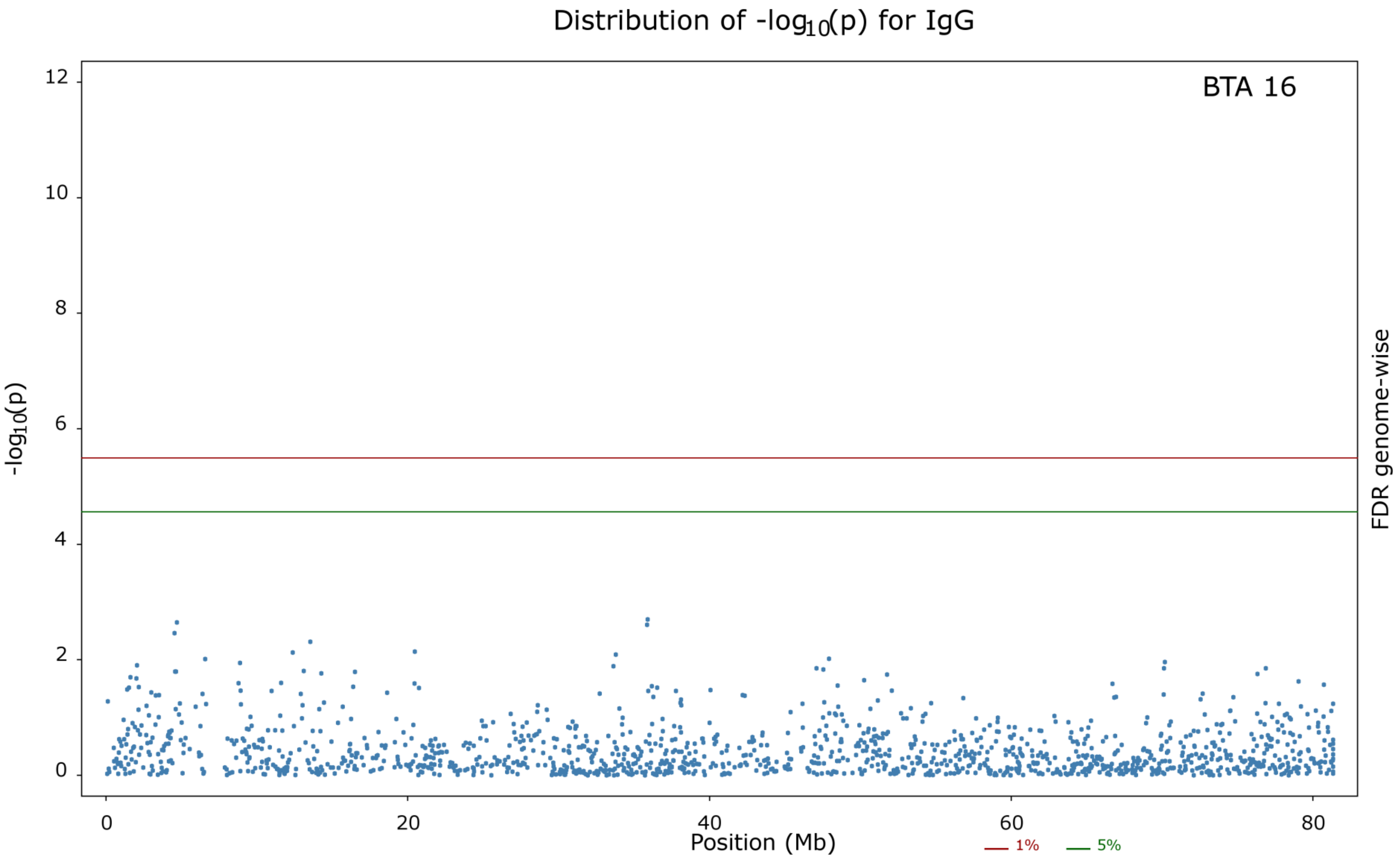
Distribution of -log10(p) for IgG
BTA 16
-log10(p)
12
10
8
6
4
2
0
0
20
40
60
80
Position (Mb)
FDR genome-wise
1%
5%

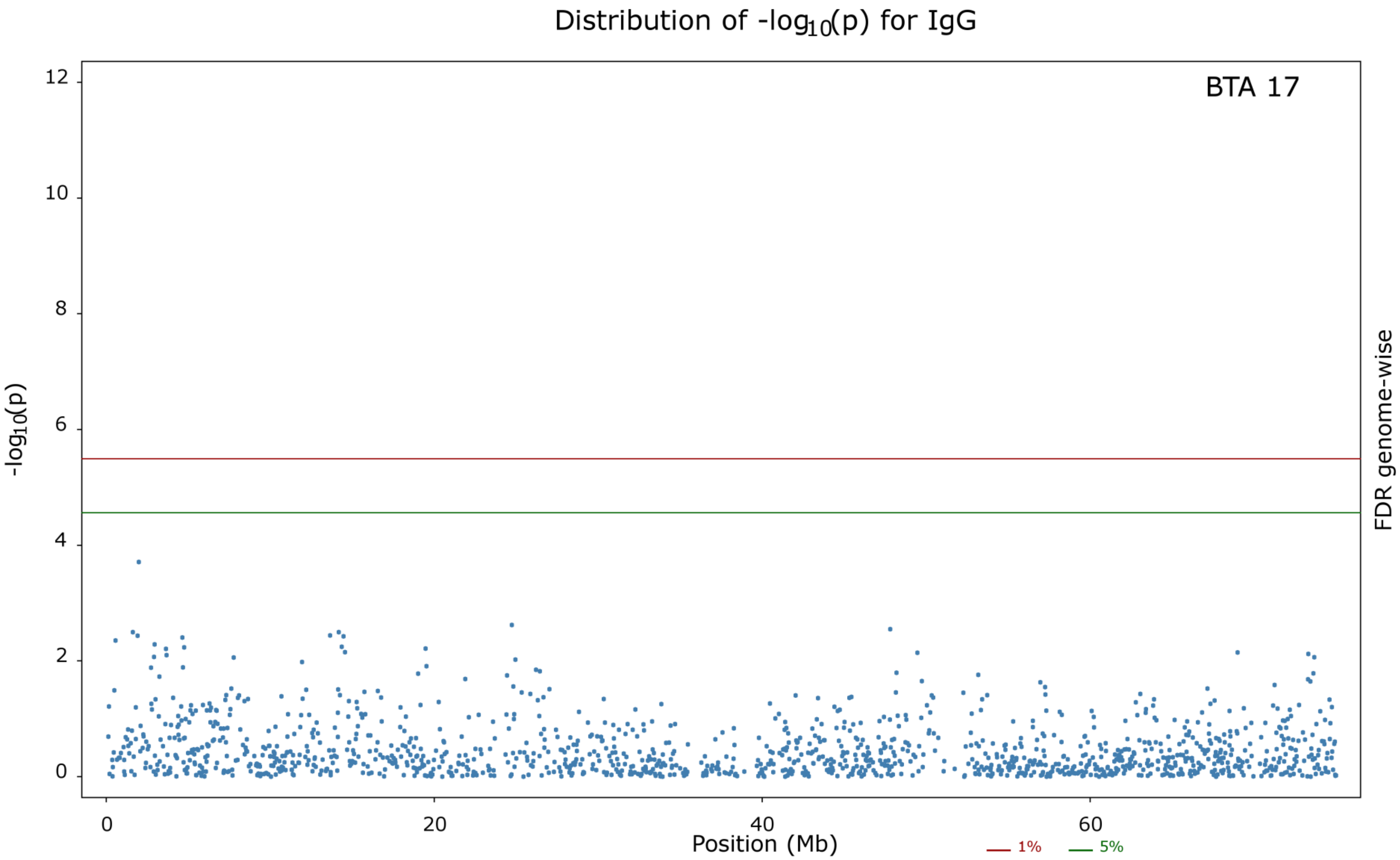

Distribution of -log10(p) for IgG
BTA 17
-log10(p)
0
2
4
6
8
10
12
0
20
40
60
Position (Mb)
FDR genome-wise
1%
5%

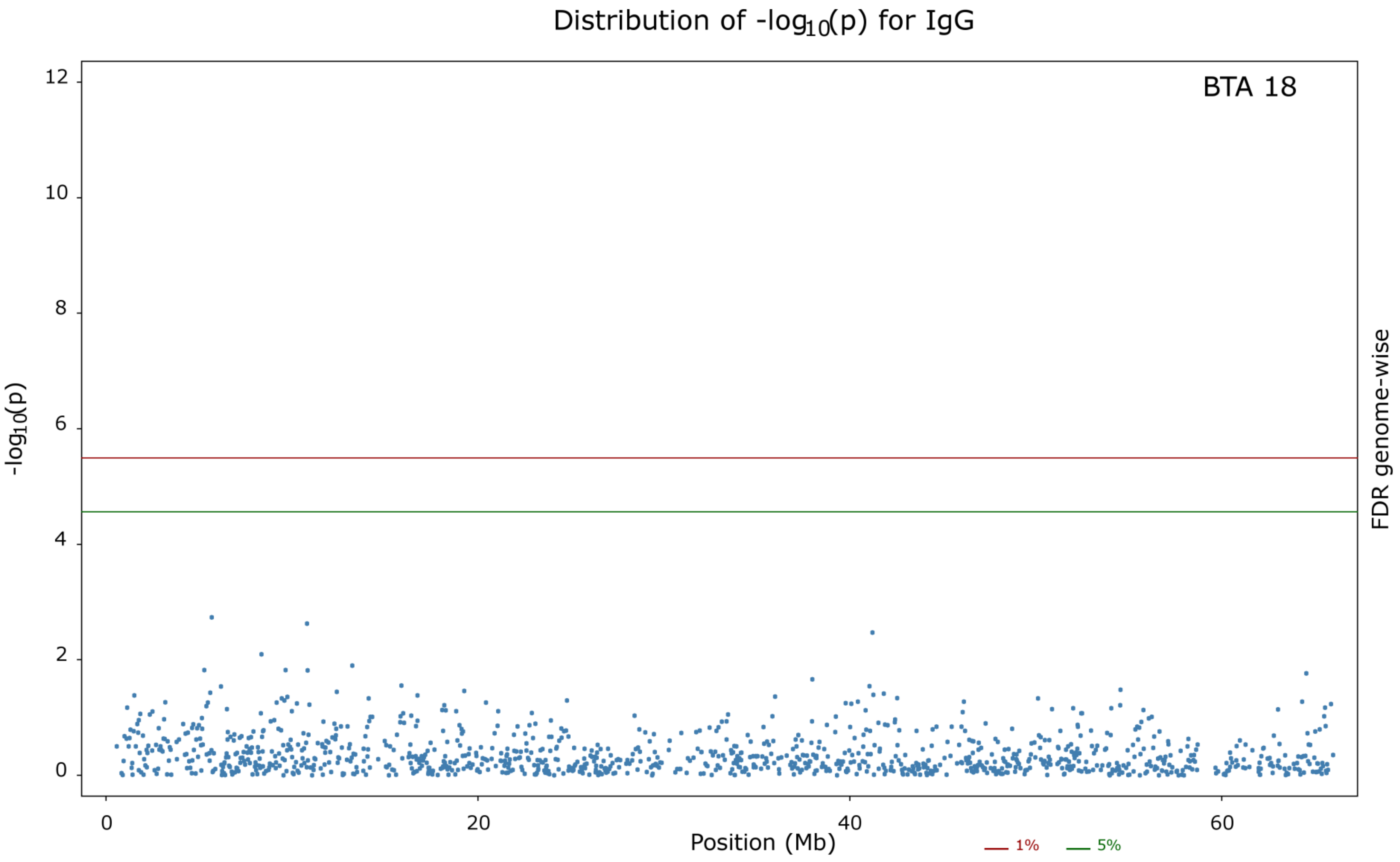

Distribution of -log10(p) for IgG
BTA 18
-log10(p)
12
10
8
6
4
2
0
0
20
40
60
Position (Mb)
FDR genome-wise
1%
5%

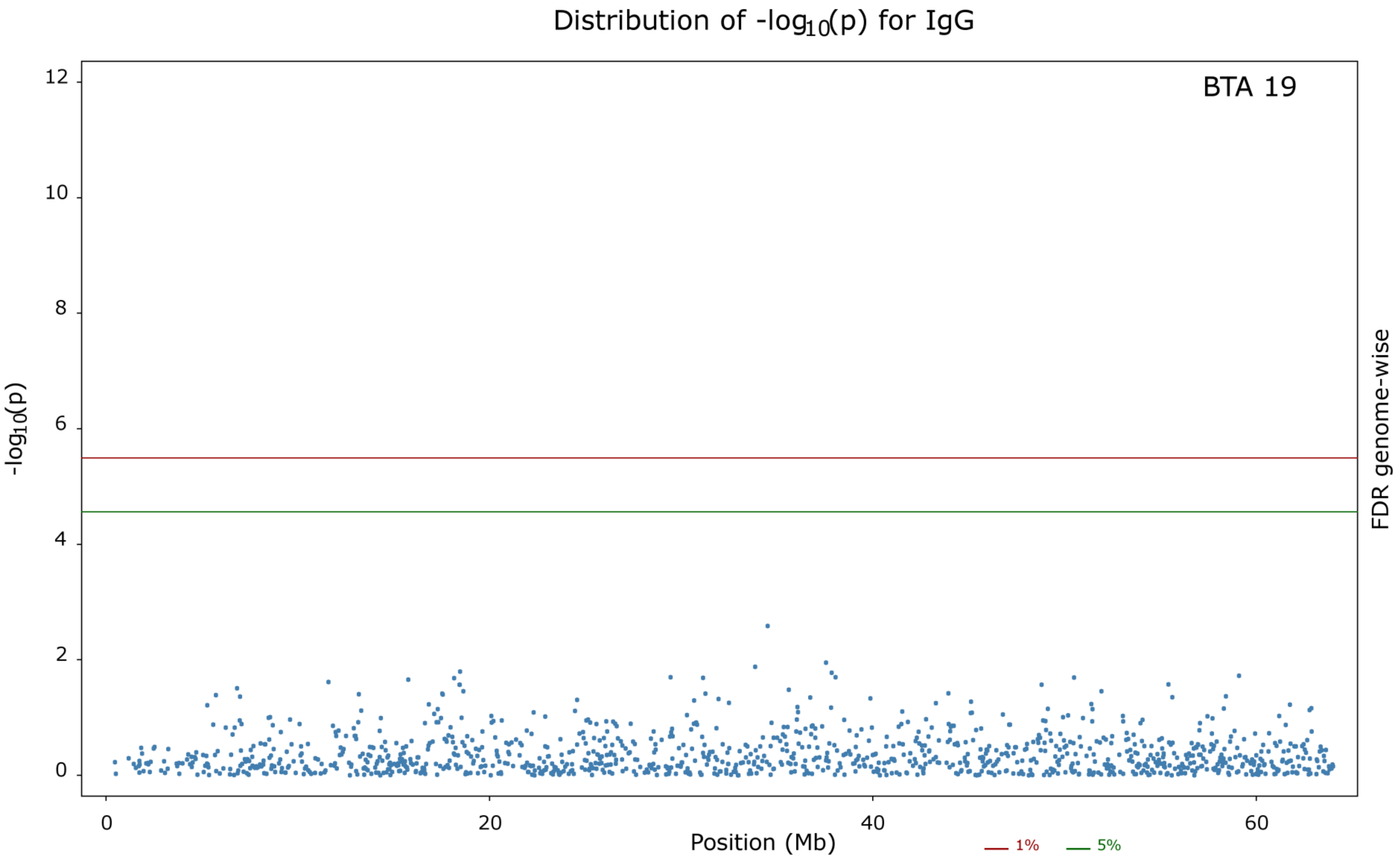

Distribution of -log10(p) for IgG
BTA 19
-log10(p)
FDR genome-wise
Position (Mb)
1%
5%
0
2
4
6
8
10
12
0
20
40
60

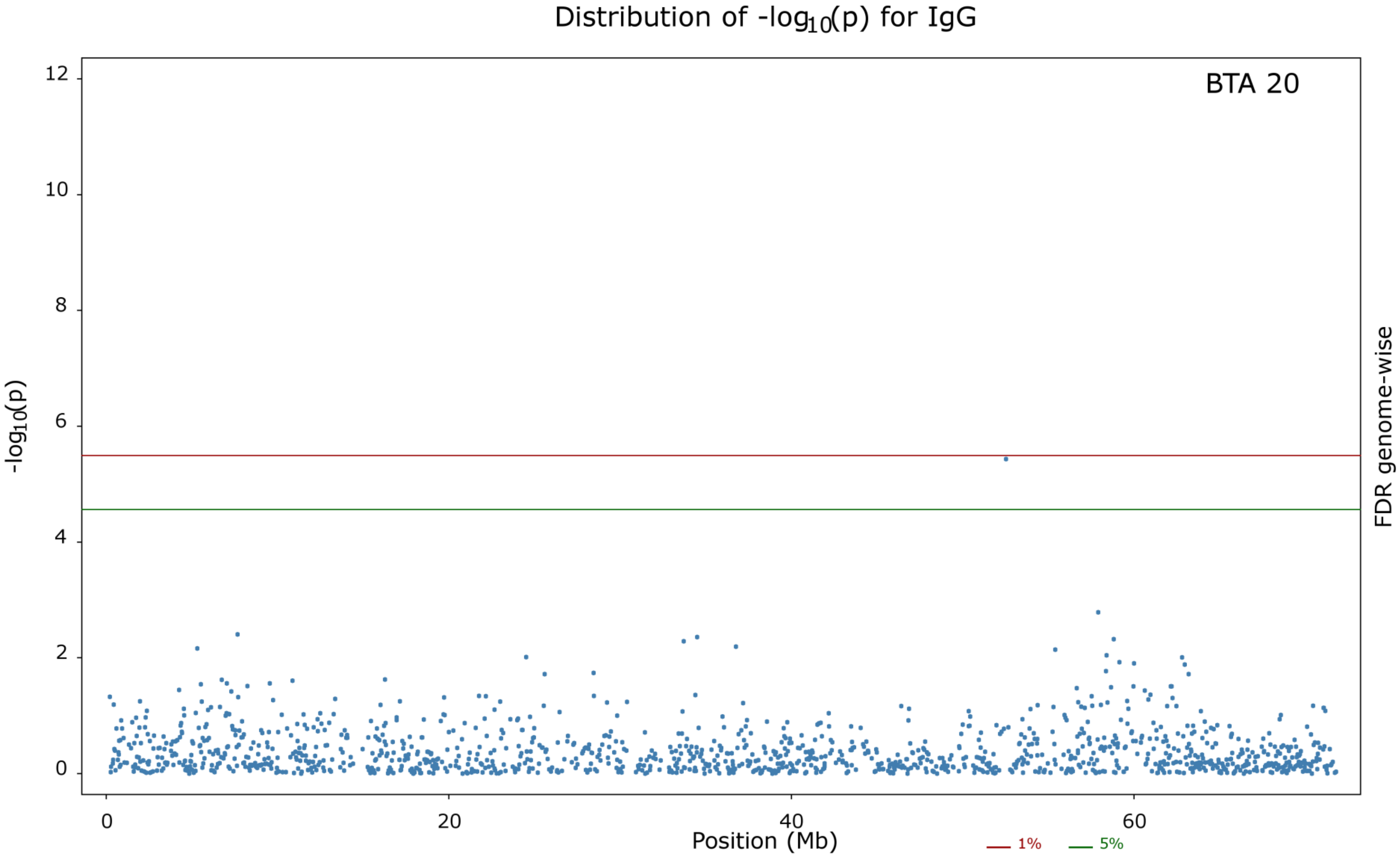

Distribution of -log10(p) for IgG
BTA 20
-log10(p)
FDR genome-wise
Position (Mb)
1%
5%
0
2
4
6
8
10
12
0
20
40
60

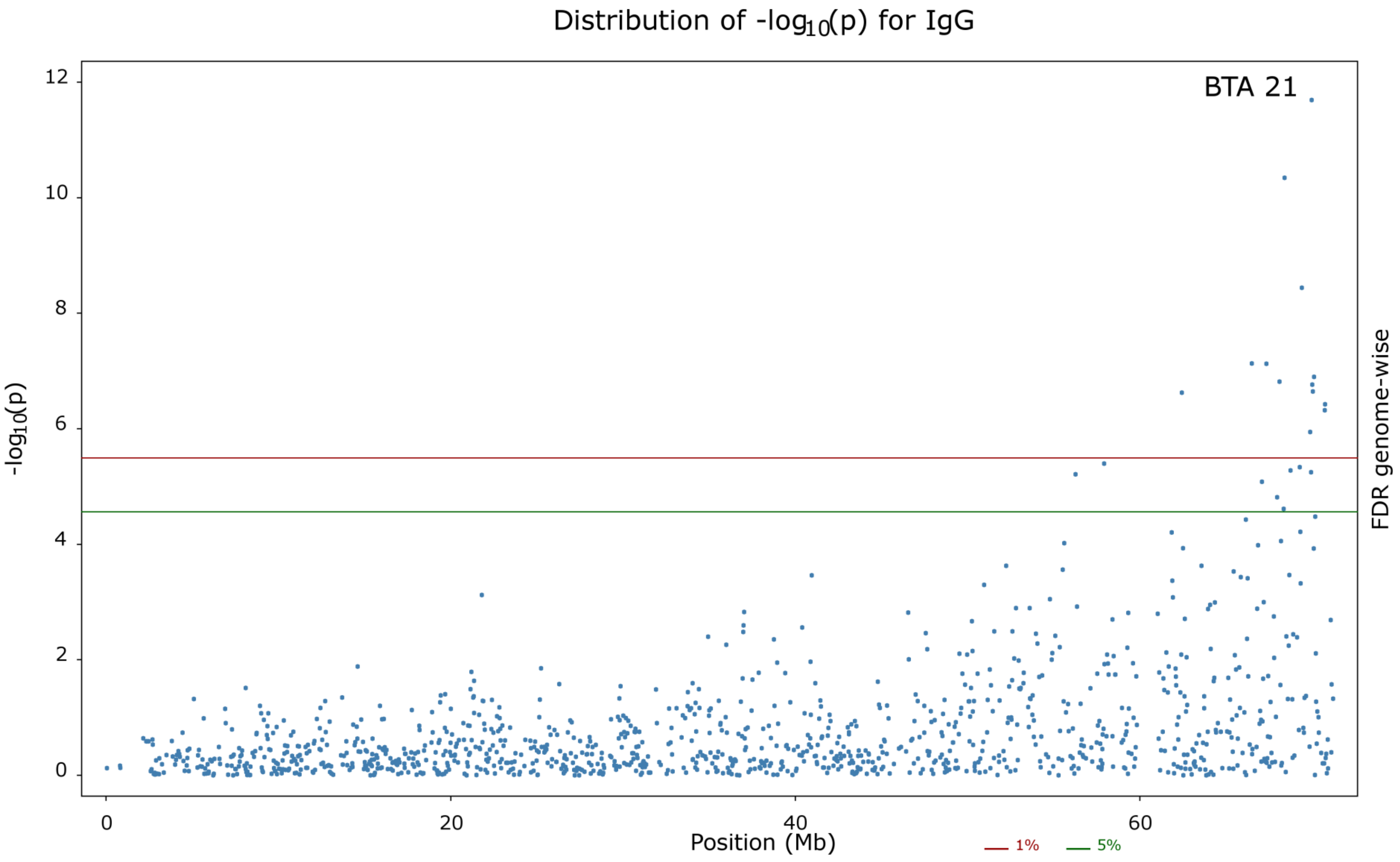

Distribution of -log$_{10}$(p) for IgG
BTA 21
-log$_{10}$(p)
FDR genome-wise
Position (Mb)
1%
5%
0
2
4
6
8
10
12
0
20
40
60

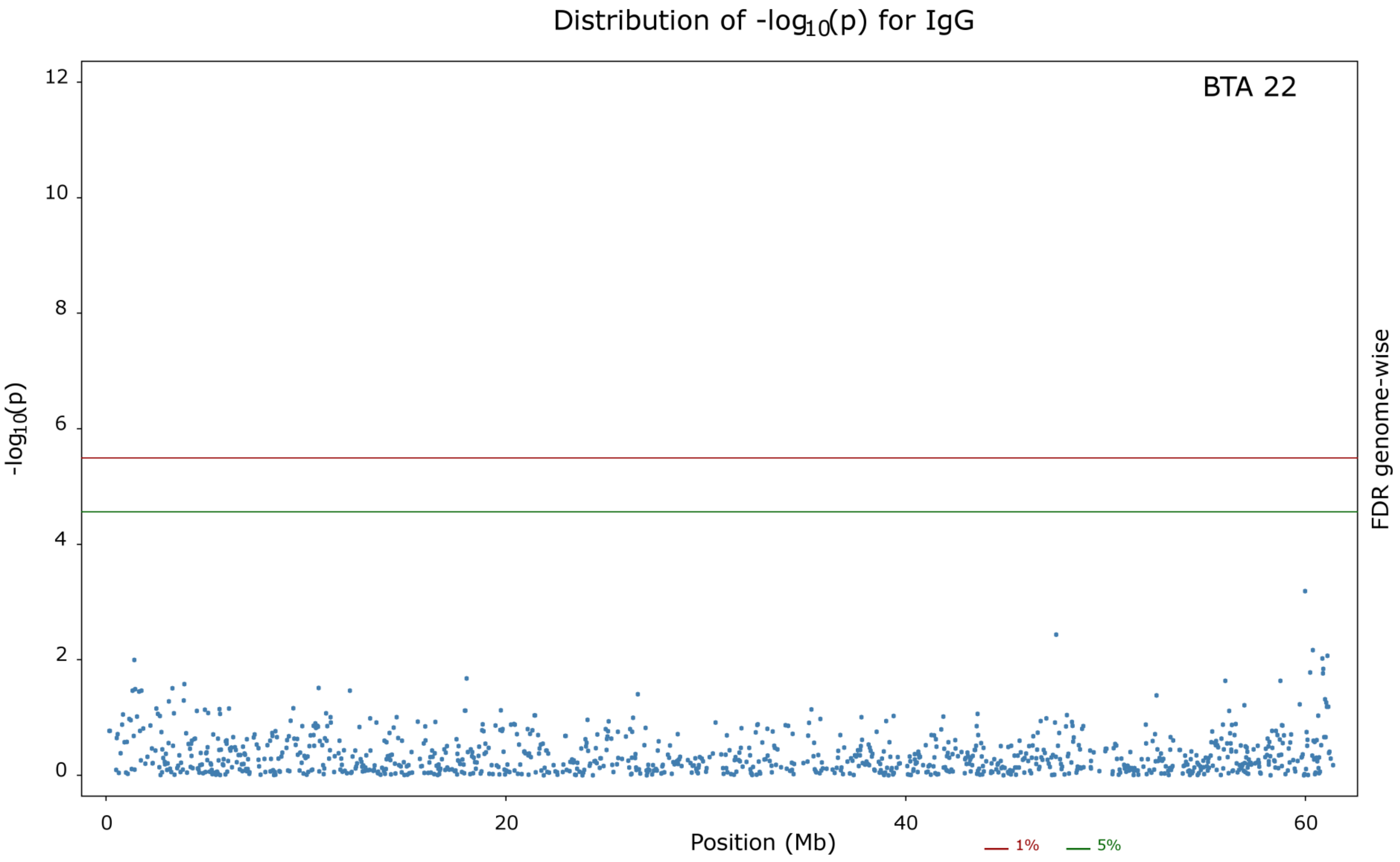

Distribution of -log10(p) for IgG
BTA 22
12
10
8
6
4
2
0
-log10(p)
FDR genome-wise
0
20
40
60
Position (Mb)
1%
5%

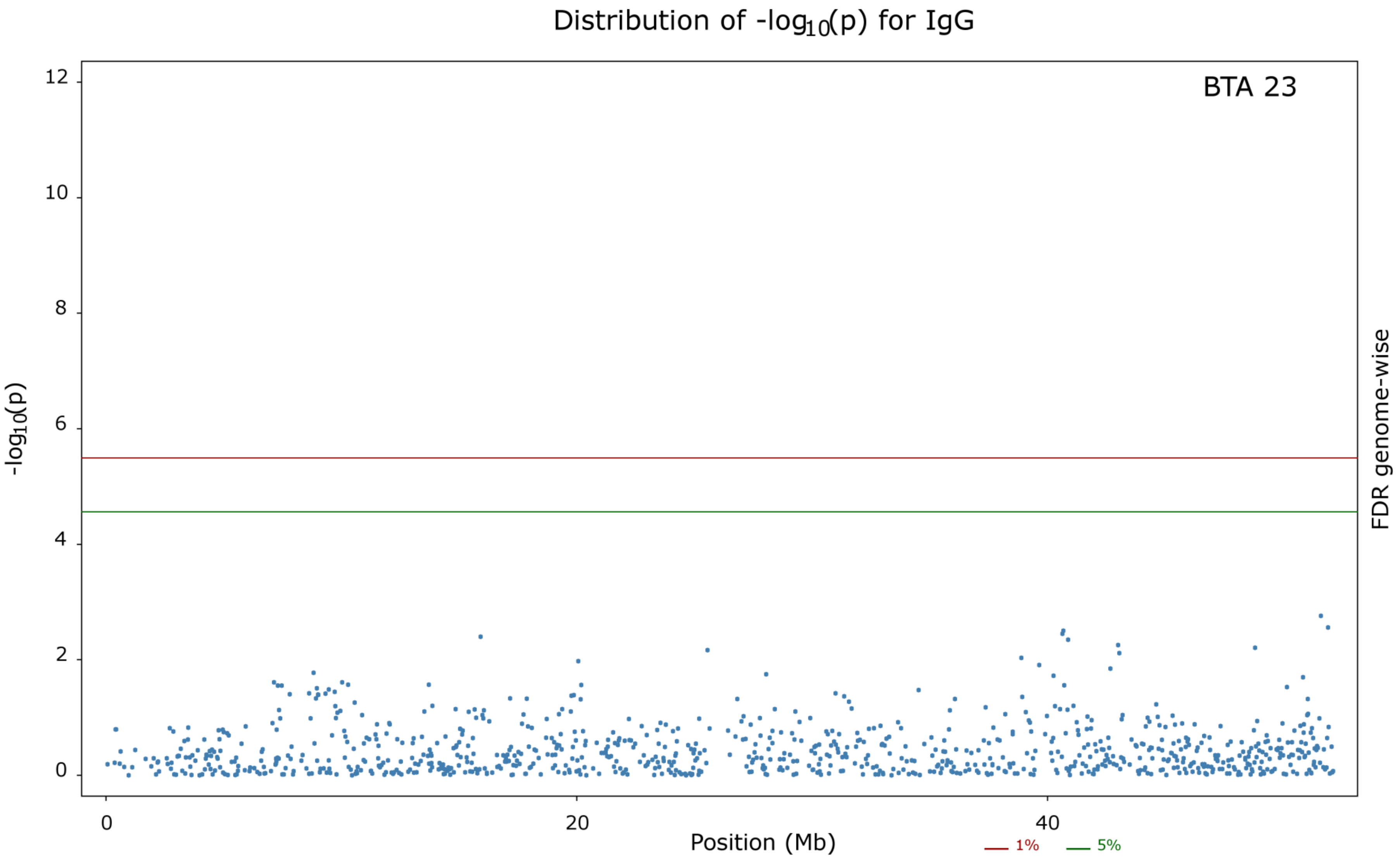

Distribution of -log10(p) for IgG
BTA 23
-log10(p)
FDR genome-wise
Position (Mb)
1%
5%
0
2
4
6
8
10
12
0
20
40

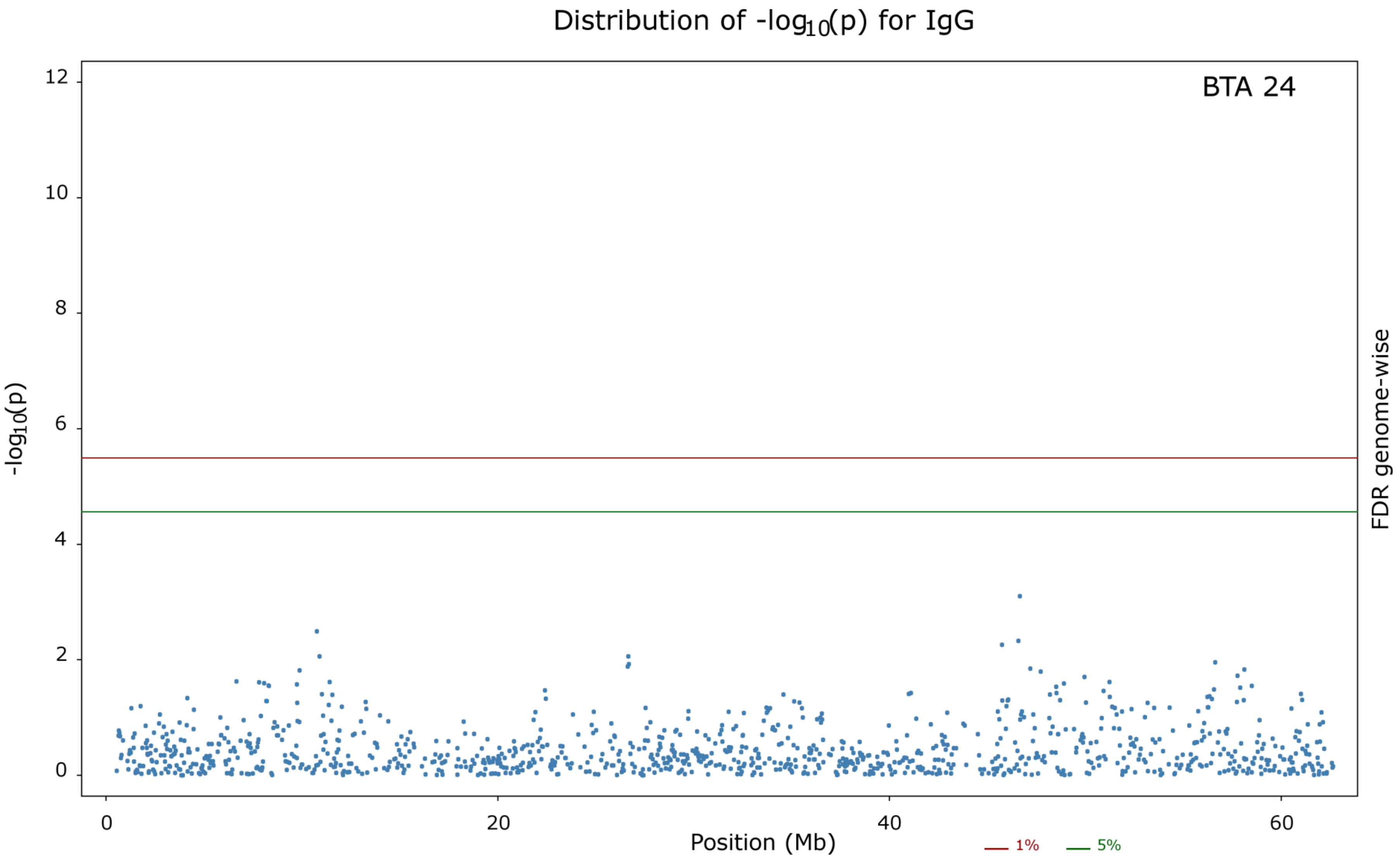

Distribution of -log$_{10}$(p) for IgG
BTA 24
-log$_{10}$(p)
0
2
4
6
8
10
12
0
20
40
60
Position (Mb)
1%
5%
FDR genome-wise

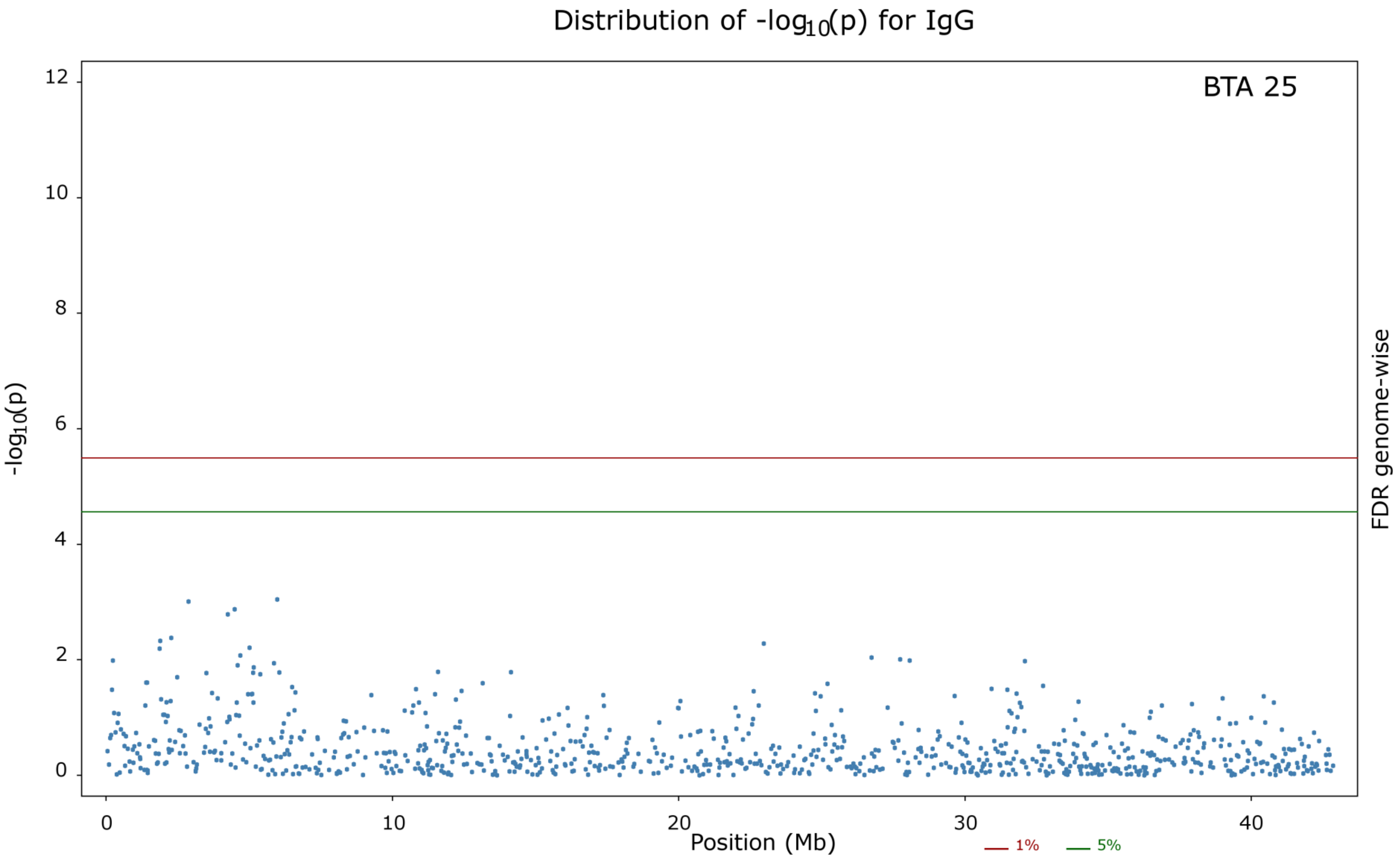

Distribution of -log10(p) for IgG
BTA 25
-log10(p)
0
2
4
6
8
10
12
Position (Mb)
0
10
20
30
40
FDR genome-wise
1%
5%

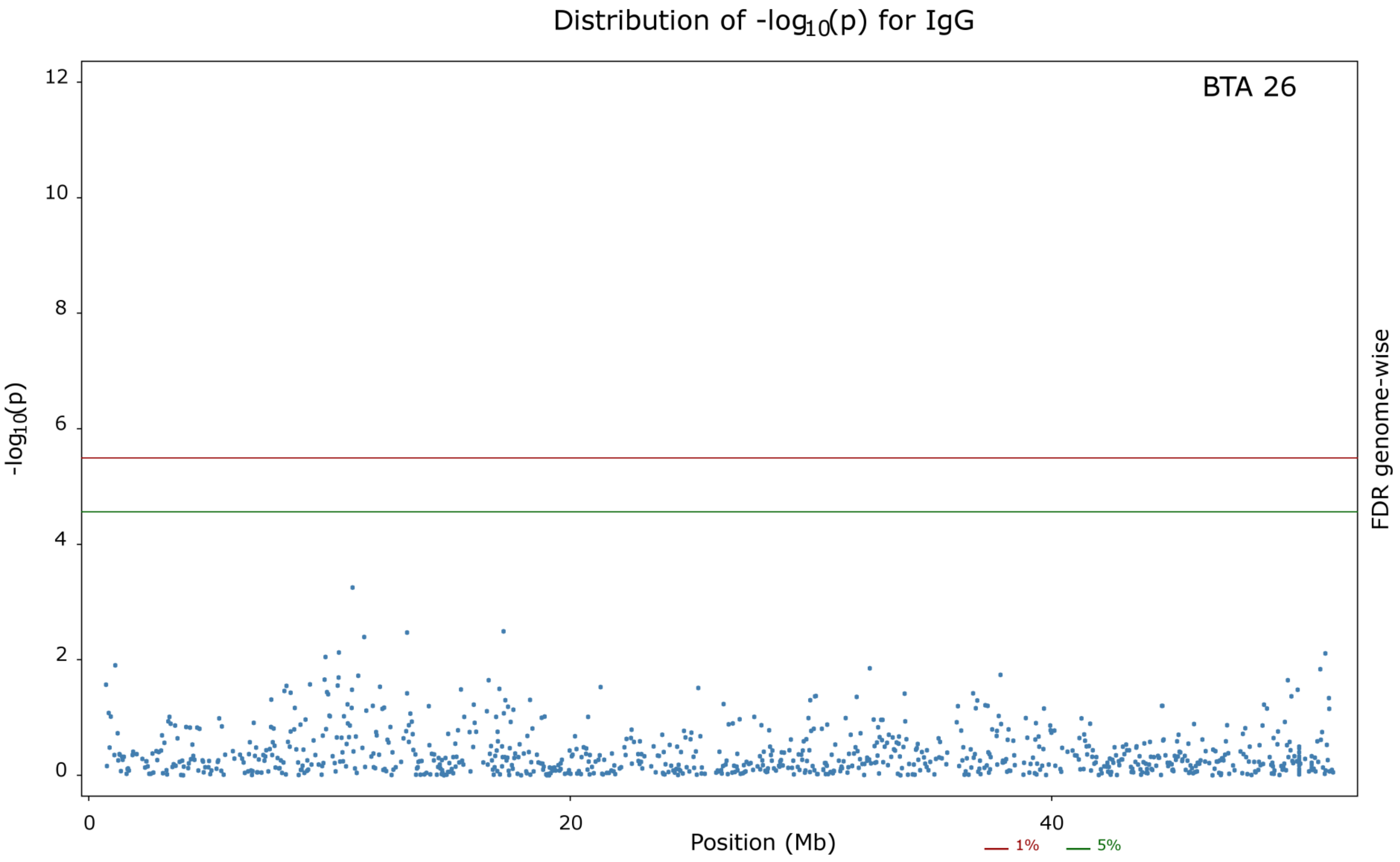

Distribution of -log10(p) for IgG
BTA 26
-log10(p)
0
2
4
6
8
10
12
0
20
40
Position (Mb)
1%
5%
FDR genome-wise

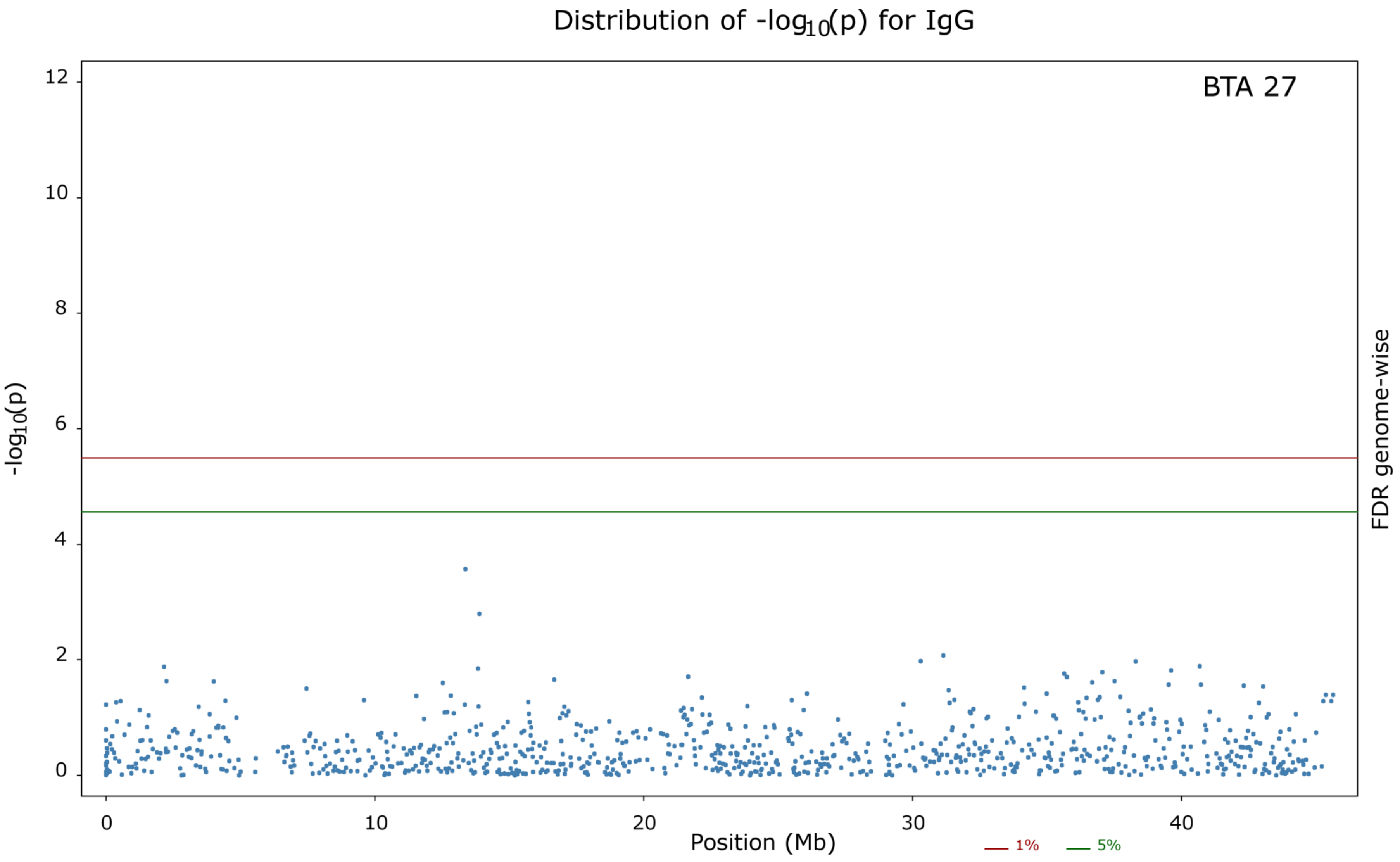

Distribution of -log10(p) for IgG
BTA 27
-log10(p)
12
10
8
6
4
2
0
0
10
20
30
40
Position (Mb)
FDR genome-wise
1%
5%

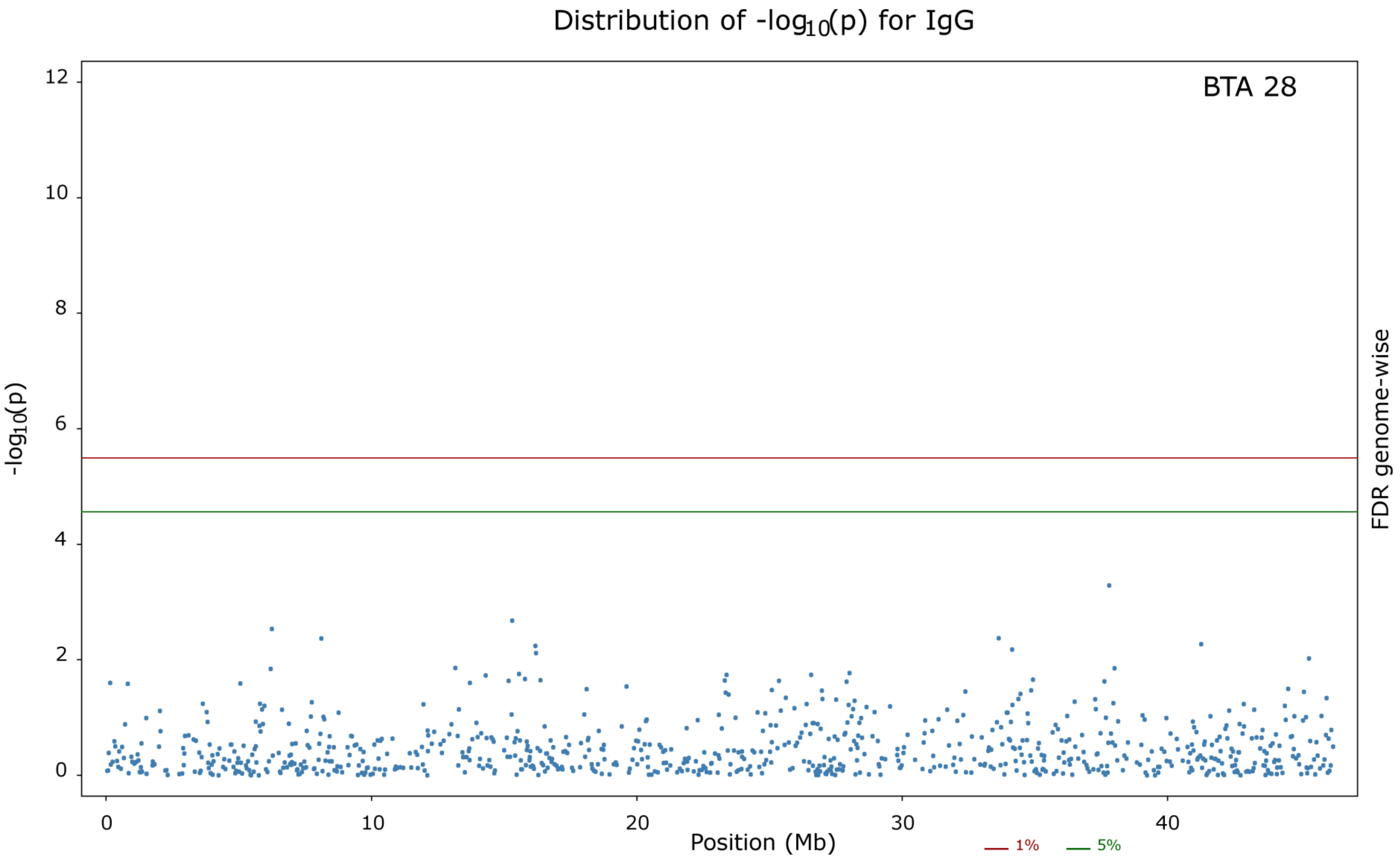

Distribution of -log10(p) for IgG
BTA 28
-log10(p)
FDR genome-wise
Position (Mb)
1%
5%
0
2
4
6
8
10
12
0
10
20
30
40

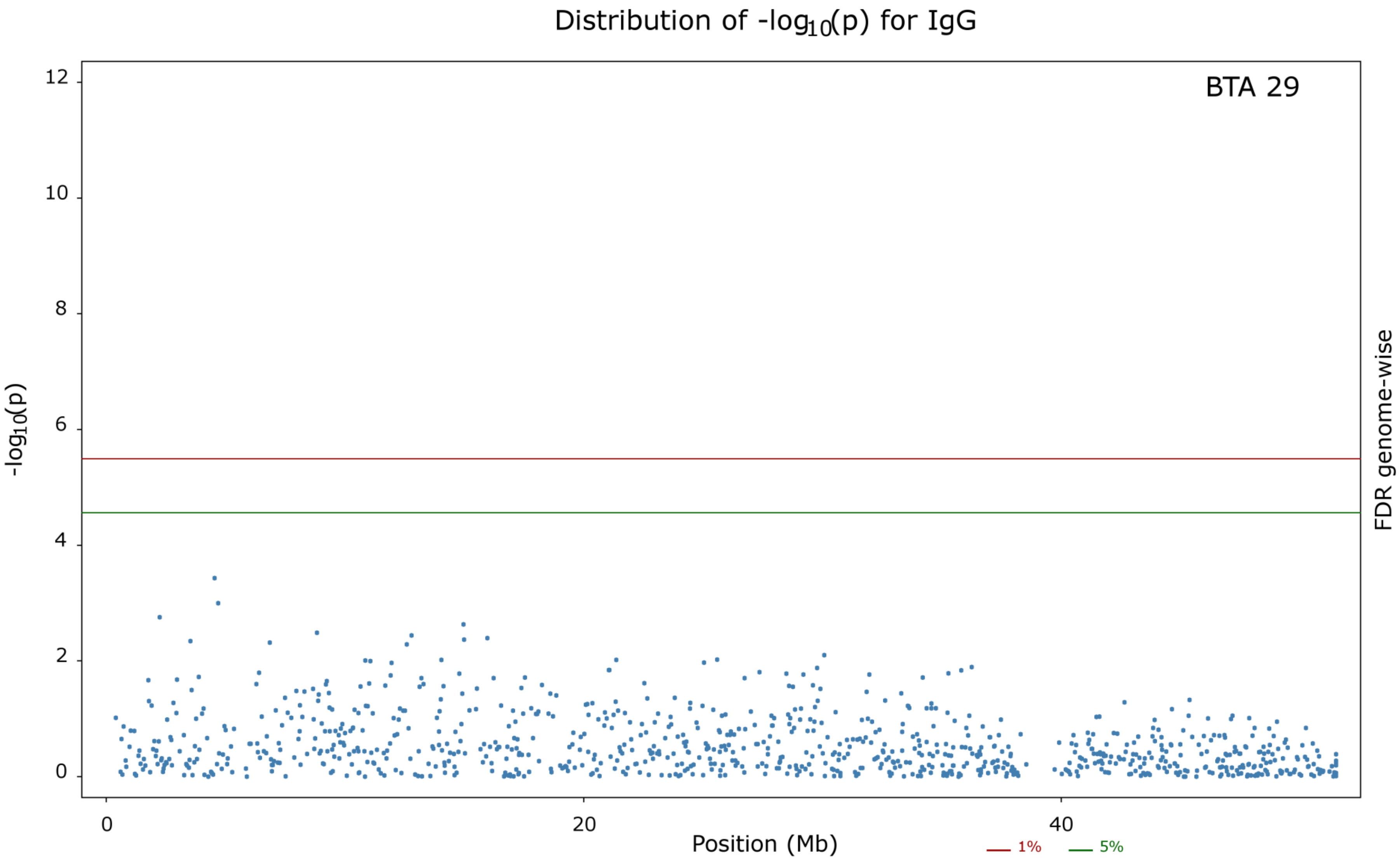

Distribution of -log10(p) for IgG
BTA 29
-log10(p)
12
10
8
6
4
2
0
0
20
40
Position (Mb)
FDR genome-wise
1%
5%
